# Time-Dependent Effect of Sciatic Nerve Injury on Rat Plasma Lipidome

**DOI:** 10.3390/ijms232415544

**Published:** 2022-12-08

**Authors:** Dmitry Senko, Anna Gorovaya, Elena Stekolshchikova, Nickolay Anikanov, Artur Fedianin, Maxim Baltin, Olga Efimova, Daria Petrova, Tatyana Baltina, Mikhail A. Lebedev, Philipp Khaitovich, Anna Tkachev

**Affiliations:** 1 Vladimir Zelman Center for Neurobiology and Brain Rehabilitation, Skolkovo Institute of Science and Technology, 121205 Moscow, Russia; 2 Research Laboratory of Mechanobiology, Institute of Fundamental Medicine and Biology, Kazan Federal University, 420008 Kazan, Russia; 3 Faculty of Mechanics and Mathematics, Moscow State University, 119991 Moscow, Russia; 4 Laboratory of Neurotechnology, I. M. Sechenov Institute of Evolutionary Physiology and Biochemistry, 194223 Saint-Petersburg, Russia

**Keywords:** lipidomics, sciatic nerve crush, blood plasma, neuropathic pain, liquid chromatography mass spectrometry, mass spectrometry, LC-MS

## Abstract

Neuropathic pain is a condition affecting the quality of life of a substantial part of the population, but biomarkers and treatment options are still limited. While this type of pain is caused by nerve damage, in which lipids play key roles, lipidome alterations related to nerve injury remain poorly studied. Here, we assessed blood lipidome alterations in a common animal model, the rat sciatic nerve crush injury. We analyzed alterations in blood lipid abundances between seven rats with nerve injury (NI) and eight control (CL) rats in a time-course experiment. For these rats, abundances of 377 blood lipid species were assessed at three distinct time points: immediately after, two weeks, and five weeks post injury. Although we did not detect significant differences between NI and CL at the first two time points, 106 lipids were significantly altered in NI five weeks post injury. At this time point, we found increased levels of triglycerides (TGs) and lipids containing esterified palmitic acid (16:0) in the blood plasma of NI animals. Lipids containing arachidonic acid (20:4), by contrast, were significantly decreased after injury, aligning with the crucial role of arachidonic acid reported for NI. Taken together, these results indicate delayed systematic alterations in fatty acid metabolism after nerve injury, potentially reflecting nerve tissue restoration dynamics.

## 1. Introduction

Neuropathic pain, defined as pain caused by nerve damage, is a condition affecting 6–10% of the population, substantially impacting their quality of life [1,2]. For research purposes, neuropathic pain is typically studied in model organisms after different types of direct peripheral nerve damage, such as ligation, transection, or compression of the sciatic nerve [3].

Nerve injury is inherently associated with a disruption in lipid composition, since they are the main constituent of important parts of the nervous system, such as the myelin sheath, and are central for proper function [4]. While the mechanisms of neuropathic pain are not fully understood, and treatment options, as well as possible biomarkers, remain limited [5], research on neuropathic pain and nerve injury from a lipidomics perspective is particularly overlooked in both humans and animal models. Nevertheless, the few studies assessing lipid alterations of nerve tissue in the peripheral nervous system following nerve injury repeatedly found increased levels of arachidonic-containing phospholipids for both rats and mice [6,7,8,9]. Omega-6 fatty acid supplementation has also been shown to assist in recovery after nerve injury [10,11]. In addition to changes in lipidome composition occurring directly at the nerve, blood lipid alterations following nerve injury, which could be related to systematic alteration of lipid metabolism, should be considered, as well. While such studies could lay groundwork for possible biomarker research, they are even more scarce [12]. At the same time, lipidomics methods have been successfully applied for the investigation of potential biomarkers of various disorders, such as cancers, neuropsychiatric disorders, and kidney and lung diseases [13,14,15], and even implemented as clinical tests in the case of cardiovascular disease [16,17].

While all contemporary lipidomics analytical methods are based on mass spectrometry, each approach has specific advantages, such as direct-infusion methods producing robust measurements and straightforward absolute quantification and reverse-phase liquid chromatography coupled to mass spectrometry (LC-MS) providing a wide coverage of lipid compounds [18]. Moreover, targeted approaches can be contrasted with untargeted methods, which, unlike the latter, do not focus on a particular set of lipid compounds beforehand. In this study, we used a comprehensive lipidomics approach based on untargeted LC-MS to investigate the lipid alterations in the blood plasma of rats following sciatic nerve crush injury. Lipid levels of injured rats were compared to those of controls at three time points: immediately after, as well as two and five weeks post sciatic nerve injury.

## 2. Results

### 2.1. Study Setup

**Figure 1:**
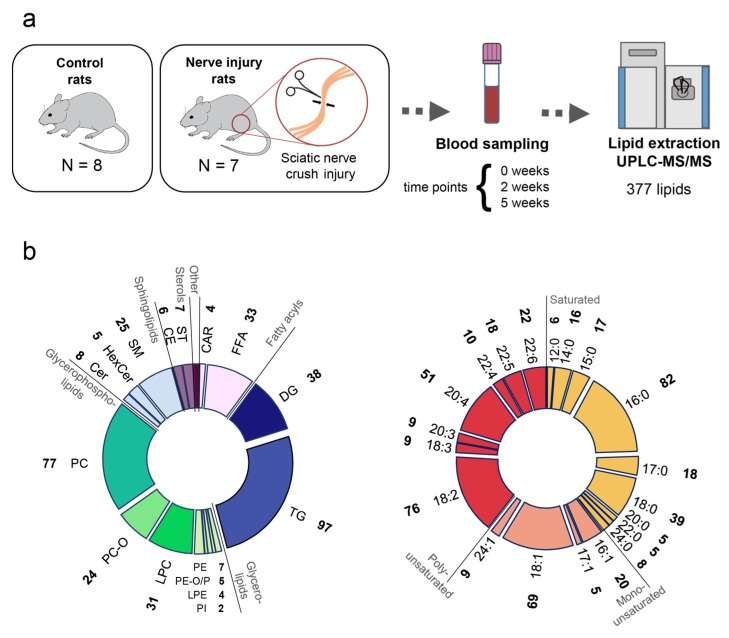
Blood lipidomics of nerve injury. (**a**) Schema of experimental design. (**b**) Distribution of quantified lipids by class (left) or fatty acid residue (right). The number of lipids in each lipid class or the number of particular fatty acid residues is indicated next to the corresponding shorthand notation. The following lipid class abbreviations are used throughout the manuscript: CAR, acylcarnitine; FFA, free fatty acid; DG, diacylglycerol; TG, triacylglycerol; PE, phosphatidylethanolamine; PE-O, plasmanylphosphatidylethanolamine; PE-P, plasmenylphosphatidylethanolamine; LPE, lysophosphatidylethanolamine; PI, phosphatidylinositol; LPC, lysophosphatidylcholine; PC-O, plasmanylphosphatidylcholine; PC, phosphatidylcholine; Cer, ceramide; HexCer, hexosylceramide; SM, sphingomyelin; CE, cholesterol ester; ST, sterol lipid.

We assessed blood plasma lipidome alterations after sciatic nerve crush injury in a time-course experiment involving seven rats with nerve injury (NI) and eight control rats (CL). The time course included three blood collection points: (i) immediately after the surgery, (ii) two weeks after the surgery, and (iii) five weeks after the surgery (w0, w2, and w5, respectively; Figure 1a). For each time point, we measured the blood plasma lipid composition using untargeted liquid chromatography coupled with mass spectrometry (LC-MS/MS). This procedure yielded mass spectrometric features assigned to 377 lipids from 17 lipid classes annotated based on their mass-to-charge values, retention times, and fragmentation patterns (Figure 1b).

**Figure 2:**
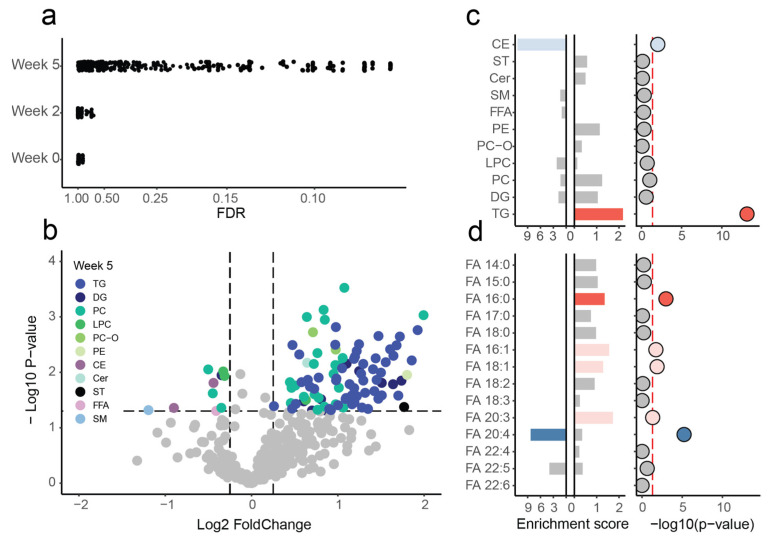
Changes in plasma lipidome detected at 5 weeks post-injury. (**a**) For the three time points, distribution of FDR-corrected *p*-values for the ANOVA test between NI and CL. Benjamini–Hochberg FDR correction was used for visualization. (**b**) Volcano plot for ANOVA test between NI and CL, with −log10 *p*-values plotted against the log2 fold change between NI and CL. NI-associated lipids are indicated in color according to lipid class, and the rest are colored in gray. (**c**,**d**) Enrichment score and −log10 *p*-values of hypergeometric test for the enrichment of lipid classes (**c**) and fatty acid (FA) residues (**d**) among NI-associated lipids. Analysis was performed separately for NI-associated lipids with increased (right-facing bar plots) and decreased (left-facing bar plots) abundances in NI. Only the lower *p*-value of the two analyses is indicated for each lipid class and fatty acid residue. Significant enrichment is marked by darker red and blue colors, and marginal enrichment is indicated by lighter shades.

At each of the three time points, we assessed the differences in abundance levels for the 377 annotated lipids between NI and CL blood plasma samples. The first and second time points showed no significant changes of the blood plasma lipidome (analysis of variance, ANOVA, permutation test *p* = 0.438 and *p* = 0.534, respectively; Figure 2a; Table S1). By contrast, at the third time point corresponding to five weeks post-surgery, we identified 106 lipids showing significant abundance alterations in NI animals (NI-associated lipids; ANOVA, *p* < 0.05 and absolute log2 fold change > 0.3; permutation test *p* = 0.0018, permutation FDR = 0.1321; Figure 2a; Table S1). A post hoc analysis of 106 NI-associated lipids at the w0 and w2 time points did not reveal any evident alteration trend correlated with w5 differences (Figure S1). An additional time-course analysis confirmed that lipidome changes took place in NI animals at w5, indicating that the detected alterations were indeed a result of the injury (Figure S2).

### 2.2. Alterations at Five Weeks Post-Injury

The NI response detected at 5 weeks post-injury showed an evident trend towards elevated levels of lipid abundances. Specifically, among the 106 NI-associated lipids, 94 displayed increased abundances in NI animals compared to CL, whereas 12 lipids were decreased in the NI group (Figure 2b; Table S3). For these two sets of lipids, we observed significant over-representation of several lipid classes, as well as an excess of particular fatty acids incorporated in the corresponding lipid structures. The increased NI-associated lipids were significantly enriched with triglyceride (TG) lipid species (hypergeometric test, *p* = 8.87 × 10^−14^, Benjamini–Hochberg corrected *p* = 4.26 × 10^−12^; Figure 2c; Table S4), as well as lipids containing palmitic (16:0) fatty acid resides (hypergeometric test, *p* = 1.07 × 10^−3^, Benjamini–Hochberg corrected *p* = 1.71 × 10^−2^; Figure 2d; Table S4). Further, there was a marginally significant enrichment of lipids containing the fatty acids 16:1, 18:1, and 20:3 in their structure (hypergeometric test, *p* = 1.91 × 10^−2^, 1.35 × 10^−2^ and 4.79 × 10^−2^, correspondingly; Figure 2d; Table S4).

The decreased NI-associated lipids, despite their low quantity, were strikingly over-represented with lipids containing arachidonic (20:4) fatty acid residues. Of the six decreased NI-associated lipids annotated at the fatty acid composition level, five had arachidonic acid in their structure (hypergeometric test, *p* = 5.93 × 10^−6^, Benjamini–Hochberg corrected *p* = 1.42 × 10^−4^; Figure 2d; Table S4). At the level of lipid classes, cholesterol esters (CE) were marginally over-represented among the decreased NI-associated lipids, as well (hypergeometric test, *p* = 1.1 × 10^−2^; Figure 2c; Table S2).

### 2.3. Interactions between Significant Lipid Classes and Fatty Acids

**Figure 3:**
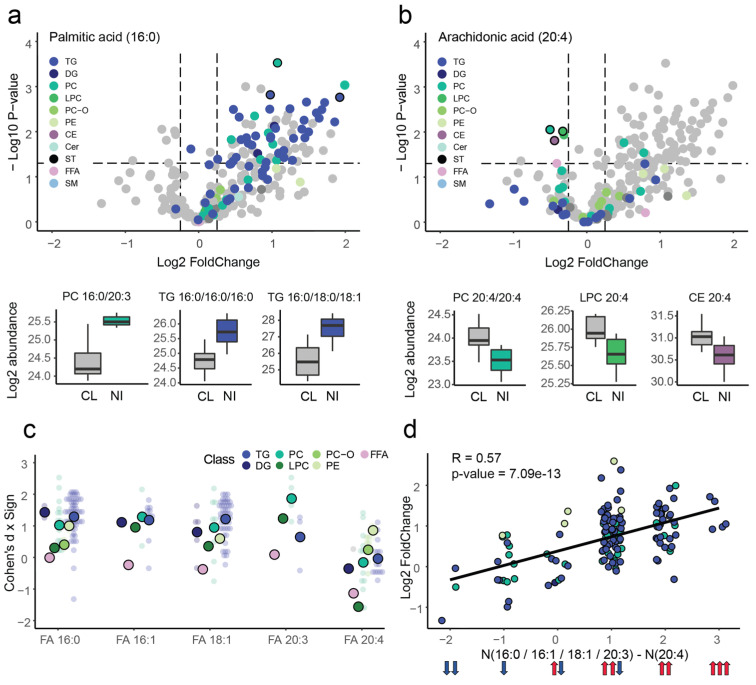
Interaction between the lipid classes and fatty acids most affected by NI. (**a**) Palmitic-acid-containing lipids. Top: volcano plot for ANOVA test between NI and CL, where lipids containing palmitic acid (16:0) are colored according to their respective lipid class. Lipids annotated at the fatty acid level but not containing palmitic acid are colored in gray. The three palmitic-acid-containing lipids with the lowest *p*-values (excluding isomers) are marked by black edges. Bottom: for these three lipids, box plots of the distributions of log2 abundances in NI and CL. (**b**) Same for arachidonic acid (20:4). (**c**) Fatty acid effects separated by lipid class. Distribution of signed Cohen’s d values for lipids containing fatty acids 16:0. 16:1, 18:1, 20:3, and 20:4, separated by lipid class (indicated by color). Smaller points correspond to a lipid, whereas bold points represent mean values for each class. (**d**) Additive effect of fatty acid residues. For a particular lipid, the number of fatty acids with negative direction of effect in NI (20:4) is subtracted from the number of fatty acids with positive direction (16:0, 16:1, 18:1, or 20:3), which is indicated on the *x*-axis, while its log2 fold changes between NI and CL is indicated on the y-axis. A linear regression was used to visualize the relationship, with correlation coefficient and corresponding *p*-value marked on the plot. The colored arrows illustrate examples of combinations of fatty acid residues with particular directions of effect in NI. All lipid classes with two or three fatty acid residues (excluding PI, *n* = 2) were considered in this analysis, i.e., PC, PE, DG, and TG. Moreover, only lipids including at least one of the affected fatty acids (16:0, 16:1, 18:1, 20:3, or 20:4) were included in the plot.

Our analysis of NI-associated lipids demonstrated separate effects related to particular lipid classes and fatty acid residues. Next, we examined the interaction between the lipid classes and fatty acids most affected by NI. We found that the over-representation of palmitic and arachidonic acid among NI-associated lipids was not limited to one particular lipid class. Indeed, NI-associated lipids from various classes contained these fatty acid residues, including TG, phosphatidylcholines (PC), and diglycerides (DG) for palmitic acid (Figure 3a), and PC, lysophosphatidylcholines (LPC), and CE for arachidonic acid (Figure 3b). Taking a closer look at the fatty acid effects separated by lipid class, we found a consistent trend, namely, an increase in NI for 16:0, 16:1, 18:1, and 20:3, together with a decrease of 20:4, for the TG, PC, LPC, and DG lipid classes (Figure 3c). This comprised four of the seven lipid classes for which this analysis was performed, i.e., those classes represented by at least five lipid species containing any of the affected fatty acids residues. Free fatty acids (FFA) showed decreased levels in NI for arachidonic acid relative to the other fatty acids, as well. On the other hand, phosphatidylethanolamines (PE) and ether phosphatidylcholines (PC-O) did not show the same general trend (Figure 3c). Moreover, while TGs, in general, were significantly increased in NI (Figure 2b,c), the TG compounds containing arachidonic acid showed a trend toward decreased abundances in NI (Figure S3). Therefore, at the fatty acid level, the effect of NI was largely consistent among multiple lipid classes, possibly indicating a systemic alteration in fatty acid metabolism.

Assessing the interaction of fatty acid residues, we noted their additive effect in NI. Specifically, the presence of multiple fatty acids with the same direction of effect enhanced abundance differences between NI and CL, while fatty acids with opposing effects canceled them out (Figure 3d and Figure S4; Pearson correlation between fatty acid composition and abundance difference between NI and CI, *c* = 0.57, *p* = 7.1 × 10^−13^). Accordingly, lipids with the strongest statistical differences between NI and CL contained multiple arachidonic acid residues, multiple palmitic acid residues, or a mixture of palmitic acid and the other three “increased” fatty acid residues, i.e., 16:1, 18:1, and 20:3. For instance, the lipid with the strongest decrease between NI and CL was found to be PC 20:4/20:4 (ANOVA, *p* = 8.86 × 10^−3^), whereas the lipid with the strongest increase between NI and CL was found to be PC 16.0_20.3 (ANOVA, *p* = 2.99 × 10^−4^) among all classes and TG 16.0/16.0/16.0 (ANOVA, *p* = 1.53 × 10^−3^) among TGs (Figure 3a,b). These results indicate that NI possibly results in alterations not just in lipid metabolism but fatty acid metabolism in particular.

## 3. Discussion

In this work, we performed a time-course experiment assessing lipid alterations in the blood plasma of rats with nerve crush injury in comparison to control animals. We detected lipidome alterations five weeks after the injury but not at earlier time points. While the literature on lipidome alterations in the blood following nerve injury is limited [12], and time-course analysis was not reported, the delayed response observed in our experiment is consistent with other types of indirect evidence. Analysis of gene expression alterations in the spinal cord following sciatic nerve injury in mice, which included time points from 0 to 8 weeks, showed variability in NI effect, with the strongest alterations observed 4 weeks post injury [19]. More generally, distinct time-dependent phases of nerve regeneration were described both at the gene expression level [20] and in terms of lipid composition [6,21], as well as nerve morphology [10,22].

At five weeks post injury, we observed a significant increase in the abundance of TGs, as well as a respective increase and decrease in abundances of lipids containing specific fatty acids: palmitic (16:0) and arachidonic acid (20:4). At the marginal level of statistical significance, we also noted alterations of other fatty acids: 16:1, 18:1, and 20:3. The study reporting alterations in the blood plasma following nerve injury [12], which included measurements in rats at three weeks post injury, was partially in line with our results. While some alterations, such as those related to ether phospholipid species, were not reproduced here, the reported lipid with the strongest decrease in abundance, CE 20:4, was among the top three decreased lipids in our study.

In nervous tissue, the most consistently reported lipidomic change following peripheral nerve injury is an increase in arachidonic-acid-containing phospholipids [6,7,8,9]. In one study of rats [6], the increase in 20:4-containing phosphatidylethanolamines reached maximum levels 30 days post injury. Since nerve myelin can incorporate fatty acids from circulation [4], and PUFAs levels such as arachidonic acid depend on the diet [23,24], a decrease of 20:4 in plasma is consistent with the increased uptake of 20:4 by the regenerating nervous system together with a limited availability pool [25,26]. Moreover, a direct impact of dietary omega-6 fatty acid intake on the level of 20:4 in peripheral nerve lipids [27], as well as the regeneration ability after injury [10], has been reported. These observations demonstrate that the increased demand for 20:4 in the nervous system is sufficiently large to possibly be reflected in the blood lipid profile, as we have found in our study. This hypothesis is also in line with our time-course results, since such changes would reflect accumulated 20:4 deficiency and would not be observed shortly after the injury. Further, an increased level of mead acid 20:3n − 9 is also a marker of 20:4 insufficiency [28].

The changes in blood lipid profile were most substantial for storage lipid classes, such as TGs and CEs, which are used to transport fatty acids to tissues [29]. Nevertheless, the effects at the fatty acid level, such as the decrease in 20:4 and increase in 16:0, 16:1, 18:1, and 20:3, were not restricted to these lipid classes. On the contrary, the fatty acid composition had a cumulative effect on the lipid abundance levels for various lipid classes: the differences between NI and CL rats was canceled out for lipids containing fatty acids with opposing effects, such as 16:0 and 20:4, and enhanced if the fatty acids had co-directional effects, such as 16:0 and 20:3.

Taken together, these results suggest systematic alterations in fatty acid metabolism following peripheral nerve injury, possibly reflecting the nerve regeneration processes that occur in the nervous tissue. While the relevance of these results with respect to human studies is exemplified by reports of the association between fatty acid levels and peripheral nerve function [30], additional studies are required to understand the implications of lipid metabolism alterations in nerve injury.

## 4. Materials and Methods

### 4.1. Animals

This study was performed using male Wistar rats weighing 150–200 g (NPK Biotech, Moscow, Russia). All animals were housed in groups of up to 8 per cage (size: 60 cm × 50 cm × 60 cm) under an artificial 12 h light–dark cycle (8:00 a.m.–8:00 p.m.) at a controlled temperature (21 ± 1 °C) and humidity (50–60%). Food and water were available ad libitum. Animal care and handling throughout the experimental procedures were in accordance with the guidelines established in Directive 2010/63/EU of the European Parliament and of the Council of 22 September 2010 on the protection of animals used for scientific purposes.

### 4.2. Sciatic Nerve Constriction Surgery and Plasma Collection

All surgical procedures were performed under anesthesia with combined injection of Zoletil 50 (Virbac, Carros, France, 50 mg/kg) and XylaVET (Pharmamagist Ltd., Budapest, Hungary, 0.05 mL/kg) administered intramuscularly to the animals.

Sciatic nerve crush was applied according to the method described by Angelis [31]. Briefly, a mosquito-type clamp was applied to anesthetized animals under aseptic conditions to the prepared sciatic nerve for 40 s. The length of the compressed area was 2 mm; the location of clamping was 1.5–2 cm above the knee joint. The wound was then sutured.

Surgical procedures and blood collection were carried out in two separate temporal batches including both control animals (CL) and rats with nerve injury (NI) (n = 4/4 and 4/3 for CL and NI in the two batches, respectively). For each batch, blood samples were collected immediately after the surgery, as well as two weeks (14 days) and five weeks (35 days) post surgery. Blood samples collected for analysis of plasma composition were taken from the right ventricle of the heart. Blood was sampled in sterile test tubes (K2EDTA, 4 mL). Tubes were stored for 10 min at 3 °C, then centrifuged for 15 min at 2000 rpm at 4 °C. Plasma samples (250–500 µL) were transferred into sterile chilled vials (Cryofreeze, 4.5 mL), frozen immediately at −30 °C, with a temperature of −78 °C for long-term storage.

### 4.3. Sample Preparation and Lipid Extraction

Extraction was performed using the protocol described in [32]. Lipid extraction was performed in a single batch in randomized order. Frozen blood plasma was kept on ice for 2 h until complete thawing. All consequent manipulations with samples were also carried out at 0 °C. A volume of 300 µL of cold (−20 °C) methanol was added to a 40 µL sample aliquot, and the mixture was shaken vigorously on a shaker for 1 min at 4 °C. Then, 1 mL of cold (−20 °C) MTBE was added, and the resulting mixture was sonicated for 10 min in an ice-cooled ultrasonic bath and incubated for 40 min at 4 °C with constant stirring, after which sonication was repeated. Then, 250 μL of chilled (4 °C) water was added to the extract, and the mixture was shaken for 1 min at 4 °C and centrifuged for 10 min at 13,000 rpm at 4 °C. An aliquot of 1000 μL of the upper layer containing non-polar components was collected in a separate vial. Then, 400 µL of the mixture of MeOH:MTBE:H_2_O = 3:10:2.5 was added to the residue for re-extraction. The sample was shaken again for 1 min at 4 °C and centrifuged for 5 min at 13,000 rpm at 4 °C. Another 300 µL of the top layer was separated and pooled, affording a 1300 μL non-polar fraction. The resulting lipid solution was evaporated to dryness in a SpeedVac centrifuge vacuum concentrator at 25 °C.

Dry lipid samples were stored at −78 °C for a several days before being re-dissolved and analyzed by UPLC-MS. Dry residue was re-dissolved in 200 µL of a cool (0 °C) mixture of ACN:IPA solution (7:3 (*v*/*v*)). The sample was shaken for 10 min, incubated in an ice-cold ultrasonic bath for 10 min, and centrifuged for 5 min at 13,000 rpm at 4 °C. Prior to UPLC/MS analysis, samples were diluted 1:5 and 1:2 with ACN:IPA solution (7:3 (*v*/*v*)) for positive and negative ionization measurements, respectively.

### 4.4. UPLC-MS Analysis

Ultraperformance liquid chromatography mass spectrometry (UPLC-MS) analyses were performed in randomized order. Analyses were performed on an QExactive mass spectrometer equipped with a heated electrospray ionization source (Thermo Fisher Scientific, Waltham, MA, USA) interfaced with a Waters Acquity UPLC chromatographic system (Waters, Manchester, UK). Liquid chromatography (LC) separation was performed using an ACQUITY UPLC BEH C8 reverse-phase column (2.1 × 100 mm, 1.7 µm, Waters Co., Milford, MA, USA) with a Vanguard pre-column with the same sorbent maintained at 60 °C. Mobile phase A consisted of 10 mM ammonium acetate in water with 0.1% formic acid, and mobile phase B consisted of 10 mM ammonium acetate in ACN:IPA (7:3) with 0.1% formic acid. For the negative detection mode, formic acid in the composition of the mobile phase was replaced by acetic acid. The flow rate was set to 0.4 mL/min in gradient elution mode according to the following program: 1 min 55% B, 3 min linear gradient from 55% to 80% B, 8 min linear gradient from 80% B to 85% B, and 3 min linear gradient from 85% B to 100% B. The phase composition was maintained at 100% B for 4.5 min, after which the column was set to initial conditions (55% B) for 4.5 min. The injection volume was 3 μL.

MS detection was carried out in positive and negative ionization modes in the mass range of *m*/*z* 100–1500 with a mass resolution of 70,000 (FWHM for *m*/*z* 200). HESI source conditions were set as follows: ion transfer tube temperature, 350 °C; vaporizer temperature, 250 °C; spray voltage, 4.5 kV; S-lens RF level, 70; AGC target value, 1 × 10^6^; sheath gas (N2) flow rate, 45 arbitrary units (a.u.); auxiliary gas (N2) flow rate, 20 a.u.; sweep gas (N2) flow rate, 4 a.u. External calibration was applied using Pierce LTQ Velos ESI positive ion calibration solution and Pierce ESI negative ion calibration solution before sample analysis to confirm mass accuracy.

To achieve a wide coverage of lipid species annotation, iterated data-dependent acquisition (DDA) approach was used. Briefly, an analytical MS/MS protocol was performed with inclusion lists containing approximately 400 *m*/*z* values for each polarity and then repeated with an exclusion list containing the same *m*/*z* values. Parameters for full MS scan mode were set for both ionization modes as follows. Resolution: 70,000 at *m*/*z* 200; AGC target: 5 × 10^5^; IT: 50 ms; mass range: 200−1800. All ions from the inclusion list within the 10 ppm range were subjected to fragmentation with following parameters. Resolution: 17,500; AGC target: 5 × 10^4^; IT: 100 ms. The intensity threshold was maintained at 8 × 10^3^, and the isolation width was set to 1.2 Da. Stepped normalized collision energy was set to 15, 20, and 25%. The dynamic exclusion parameter was set to 12 s. Data was acquired in profile mode, the peptide match option was off, and isotope exclusion was set to on.

Technical samples were analyzed together with the plasma samples. Empty tubes without plasma (extractions blanks) were placed at the end of the experiment and subjected to the same analysis steps as plasma samples. Quality control samples (QC) were incorporated every 12th position to account for sample preparation and measurement variability and were also used for MS/MS analysis. QC samples consisted of aliquots of plasma, mixed after extraction.

### 4.5. Data Processing and Analysis

UPLC-MS spectra were converted from raw to ABF format using an ABF converter. Then, files were processed using MS-DIAL software (version 4.70) [33]. Parameters were set as follows: MS1 tolerance, 0.05; minimum peak height, 10,000; mass slice width, 0.05. The integrated MS-DIAL lipid database was used for annotation (Table S1). All annotations reported by MS-DIAL were additionally verified by manual inspection of their MS/MS spectra. A full list of MS-DIAL parameters used in the analysis is presented in Table S1. Blank filtration was performed in MS-DIAL (Table S1), and all zero values were replaced by 1/5 of the feature minimal value. Results were exported from MS-DIAL as a “raw data area” matrix. Polarities were manually combined to include uniquely annotated compounds. Additionally, lipid features were filtered according to their variability in TQC samples, and all features with a coefficient of variance > 0.4 were removed. Lipid feature abundances were transformed using base-2 logarithm. The experimental sample batch effect was corrected by batch mean subtraction for each lipid. As a result, a table of abundances for 377 lipid compounds annotated by MS/MS was produced. The full list of annotated lipids is available in Table S2. Lipids were classified according to the nomenclature provided by LIPID MAPS [34].

Statistical analyses and data visualization were performed in R [35,36]. For each time point separately, analysis of variance (ANOVA) was used to assess differences between NI and CL. Lipids with a *p*-value < 0.05 and absolute difference of mean log2 abundances in NI and CL (absolute log2 fold-change) > 0.3 were considered significant. The false-discovery rate (FDR) for significant lipids and overall statistical effect were assessed by permutation procedures. Specifically, for each time point, sample labels were randomized 10,000 times. Then, the mean number of significant lipids for the 10,000 permutations was used as the FDR estimate. To calculate the permutation *p*-value, we assessed the proportion of permutations for which there was more or an equal number of significant lipids than the true number of significant lipids.

To study the over-representation of lipid classes and fatty acid residues among NI-associated lipids, we used a hypergeometric test. The test was applied separately for NI-associated lipids with increased and decreased abundances in NI compared to CL, defined according to their difference of mean log2 abundances (log2 fold change) between groups. For lipid classes, the number of lipid species from the corresponding lipid class was considered in the enrichment analysis. For fatty acid residues, the number of times the fatty acid was found in the structure of a lipid, including multiple occurrences of the same fatty acid in a compound, was considered in the enrichment analysis, discarding lipids without annotation at the fatty acid composition level. Sphingoid bases, such as 18:1;O2 for Cer 18:1;O2/18:1, and ether-linked fatty acids, such as O-18:1 for PC O-18:1/16:0, were disregarded in this analysis. The hypergeometric test *p*-values for lipid classes and fatty acid residues were corrected for multiple testing using Benjamini–Hochberg correction, separately for NI-associated lipids decreased and increased in nerve injury. Fatty acids and lipid classes with a corrected *p*-value < 0.05 were considered significantly enriched, whereas those with a nominal *p*-value < 0.05 were considered marginally significant. For lipid classes, enrichment scores were calculated as follows: $N\left(class,sign\right)/Nclass\frac{Nsign}{Ntotal}$, where *Ntotal* is the total number lipids in the analysis, *Nsign* is the number of NI-associated lipids with increased or decreased abundances, *Nclass* is the number of lipids in the lipid class, and *N*(*class*,*sign*) is the number of lipids in the intersection of the latter two groups. For fatty acid residues, enrichment scores were calculated similarly by counting fatty acid residues instead of lipids.

## 5. Conclusions

In this work, we assessed changes in the rat plasma lipidome after sciatic nerve crush injury, an often-used model of neuropathic pain. Blood lipids were measured at three time points: immediately after, as well as two and five weeks post injury. Of the three time points, only the latter showed significant blood plasma lipid alterations between rats with nerve injury and controls. These alterations involved lipids with particular chemical properties: triglycerides and lipids containing specific fatty acid residues, i.e., 20:4, 16:0, 16:1, 18:1, and 20:3, of which the former two fatty acid residues showed strongest statistical effects. These results indicate delayed systematic changes in lipid metabolism potentially associated with post-injury nerve tissue recovery.

## Data Availability

Raw data materials are available upon request.

## Supplementary Materials

The following supporting information can be downloaded at: https://www.mdpi.com/article/10.3390/ijms232415544/s1.

## Abbreviations

ACN	acetonitrile
CAR	acylcarnitine
Cer	ceramide
CE	cholesterol ester
DG	diacylglycerol
FFA	free fatty acid
HexCer	hexosylceramide
IPA	isopropyl alcohol
LPC	lysophosphatidylcholine
LPE	lysophosphatidylethanolamine
PC	phosphatidylcholine
PC-O	plasmanylphosphatidylcholine
PE	phosphatidylethanolamine
PE-O	plasmanylphosphatidylethanolamine
PE-P	plasmenylphosphatidylethanolamine
PI	phosphatidylinositol
SM	sphingomyelin
ST	sterol lipid
TG	triacylglycerol
