# Supplementary material for: Time-Dependent Effect of Sciatic Nerve Injury on Rat Plasma Lipidome
Source: Int J Mol Sci. 2022 Dec 8;23(24):15544. doi: 10.3390/ijms232415544 (PMC9778848; doi:10.3390/ijms232415544)
Supplement: Supplementary file 1 [file ijms-23-15544-s001.zip › ijms-2035334-supplementary.pdf]

# Time-Dependent Effect of Sciatic Nerve Injury on Rat Plasma Lipidome

**Dmitry Senko [1], Anna Gorovaya [1], Elena Stekolshchikova [1], Nickolay Anikanov [1], Artur Fedianin [2], Maxim Baltin [2], Olga Efimova [1], Daria Petrova [1], Tatyana Baltina [2], Mikhail A. Lebedev [3,4], Philipp Khaitovich [1] and Anna Tkachev [1,*]**

[1] Vladimir Zelman Center for Neurobiology and Brain Rehabilitation, Skolkovo Institute of Science and Technology, 121205 Moscow, Russia
[2] Research Laboratory of Mechanobiology, Institute of Fundamental Medicine and Biology, Kazan Federal University, 420008 Kazan, Russia
[3] Faculty of Mechanics and Mathematics, Moscow State University, 119991, Moscow, Russia
[4] Laboratory of Neurotechnology, I. M. Sechenov Institute of Evolutionary Physiology and Biochemistry, 194223 Saint-Petersburg, Russia
* Correspondence: a.tkachev@skoltech.ru

## Content:

**Figure S1.** Analysis of 106 NI-associated lipids at the w0 and w2 time points.

**Figure S2.** Changes in lipid abundances from the first time point to the later ones for the NI and CL groups, separately.

**Figure S3.** Effect of fatty acid composition on triglyceride abundances in NI at w5.

**Figure S4.** Additive effect of fatty acid residues, shown separately by lipid class.

**Table S1.** MS-DIAL parameters of data processing

**Table S2.** Full list annotated lipids.

**Table S3.** List of *p*-values and log2 fold changes at the three time points for 377 lipids.

**Table S4.** Enrichment analysis results for lipid classes and fatty acid residues.

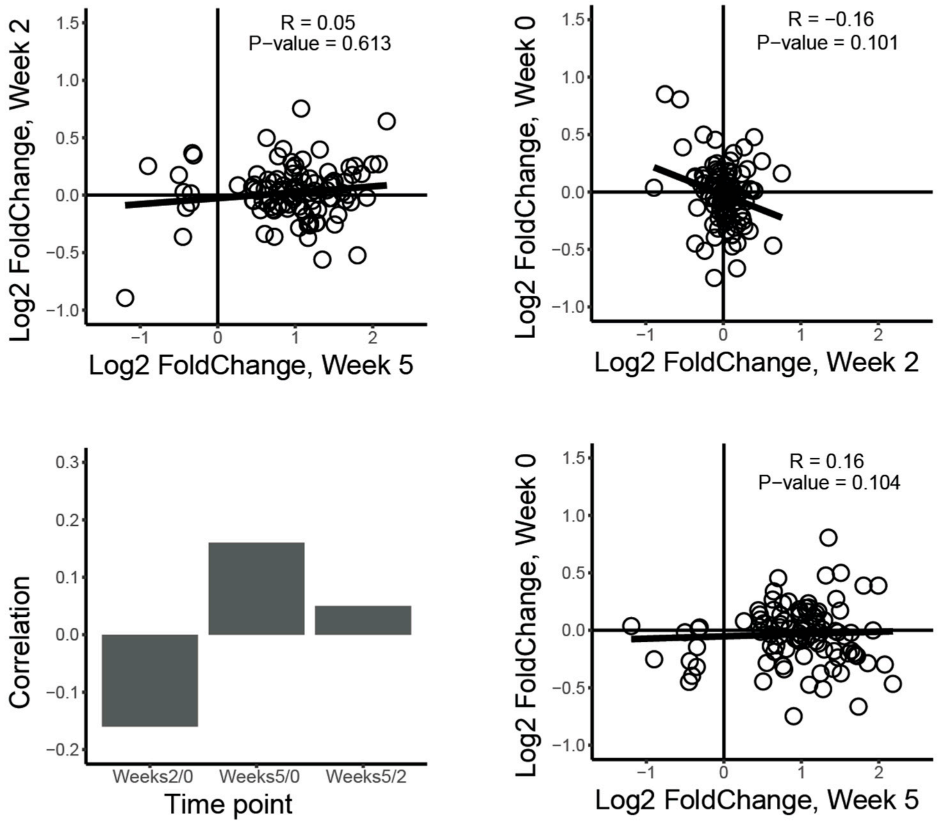


**Figure S1.** Analysis of 106 NI-associated lipids at the w0 and w2 time points. Here are shown the scatter plots of log2 fold changes between NI and CL at the different time points. The Pearson correlation cofficients and corresponding *p*-values are included on the plots. No evident alteration trend correlated with w5 alterations was found at w0 and w2.

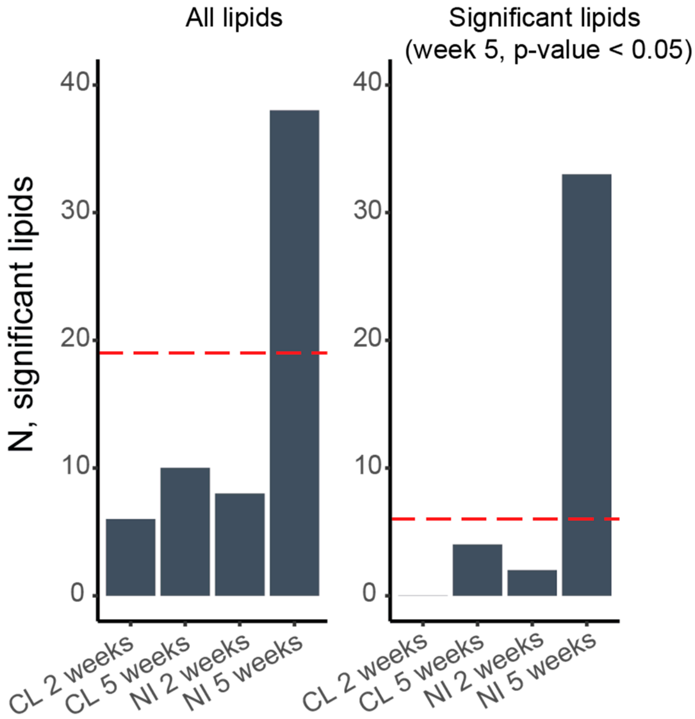


**Figure S2.** Changes in lipid abundances from the first time point to the later ones for the NI and CL groups, separately. Here are shown the number of lipids with significant (paired t-test, nominal $p$-value < 0.05) changes in abundance between weeks 0/2 or 0/5 for CL and NI groups. The analysis was performed for all lipids (left) and only NI-associated lipids (right). The red line indicates the expected number of significant lipids under a uniform $p$-value distribution (total number of lipids × 0.05). Changes in the NI group between week 0 and 5 were most pronounced among all other comparisons.

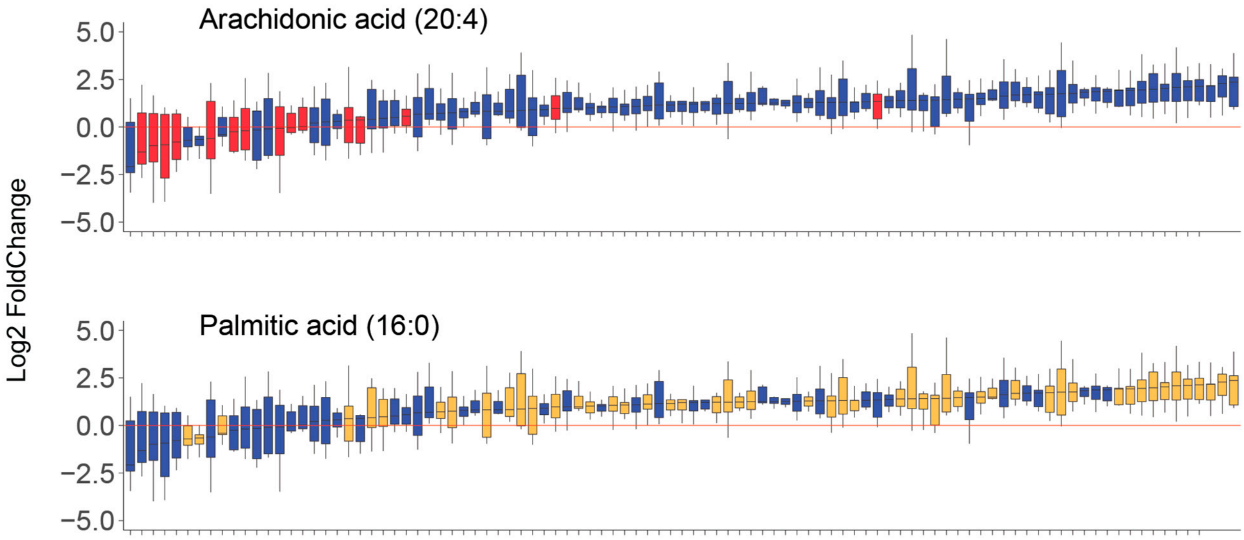


**Figure S3.** Effect of fatty acid composition on triglyceride abundances in NI at w5. For each triglyceride, the boxplot indicates the distribution of the differences between log2 abundances in NI and the mean log2 abundance in CL. TG features are ordered by boxplot medians. Top: TG containing arachidonic acid are colored in red. Bottom: TG containing palmitic acid are colored in yellow.

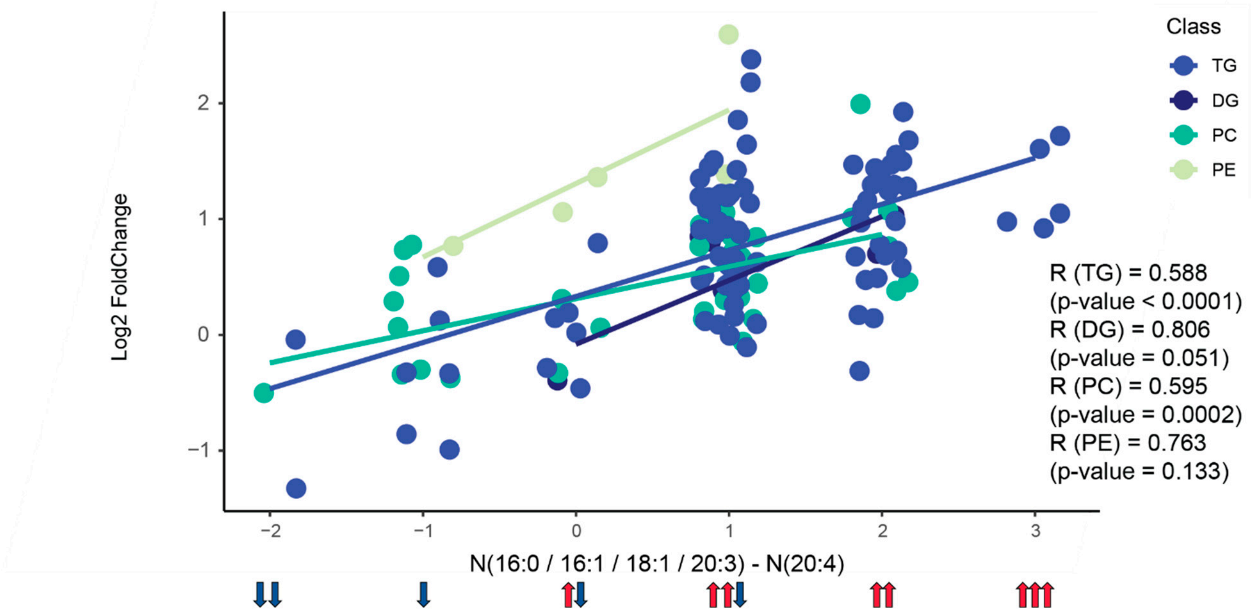


**Figure S4**. Additive effect of fatty acid residues, shown separately by lipid class. For a particular lipid, the number of fatty acids with negative direction of effect in NI (20:4) is subtracted from the number of fatty acids with positive direction (16:0, 16:1, 18:1, or 20:3), which is indicated on the *x*-axis, while its log2 fold changes between NI and CL is indicated on the y-axis. All lipid classes with 2 or 3 fatty acid residues (excluding PI, n = 2), were considered in this analysis: PC, PE, DG, TG. Moreover, only lipids including at least one of the affected fatty acids (16:0, 16:1, 18:1, 20:3, 20:4) are included in the plot. For each lipid class separately, a linear regression was used to visualize the relationship, with correlation coefficient and corresponding *p*-value marked on the plot.

**Table S1**. MS-DIAL parameters of data processing.

| **MS-DIAL ver. 4.70** | | |
|---|---|---|
| #Project | | |
| MS1 Data type | Profile | |
| MS2 Data type | Profile | |
| Ion mode | Positive/Negative | |
| Target | Lipidomics | |
| Mode | ddMSMS | |
| #Data collection parameters | | |
| Retention time begin | | 1 |
| Retention time end | | 17 |
| Mass range begin | | 200 |
| Mass range end | | 1200 |
| MS2 mass range begin | | 50 |
| MS2 mass range end | | 1200 |
| #Centroid parameters | | |
| MS1 tolerance | | 0.01 |
| MS2 tolerance | | 0.025 |
| #Isotope recognition | | |
| Maximum charged number | | 2 |
| #Data processing | | |
| Number of threads | | 5 |
| #Peak detection parameters | | |
| Smoothing method | LinearWeightedMovingAverage | |
| Smoothing level | | 3 |
| Minimum peak width | | 5 |
| Minimum peak height | 10000 (positive) / 20000 (negative) | |
| #Peak spotting parameters | | |
| Mass slice width | | 0.1 |
| #Deconvolution parameters | | |
| Sigma window value | | 0.5 |
| MS2Dec amplitude cut off | | 0 |
| Exclude after precursor | True | |
| Keep isotope until | | 0.5 |
| Keep original precursor isotopes | False | |
| #MSP file and MS/MS identification setting | | |
| MSP file | LipidMsmsBinaryDB-VS68-FiehnO | |
| Retention time tolerance | - | |
| Accurate mass tolerance (MS1) | | 0.01 |
| Accurate mass tolerance (MS2) | | 0.05 |
| Identification score cut off | | 80 |
| Using retention time for scoring | False | |
| Using retention time for filtering | False | |
| #Selected lipid types | | |
| All | | |
| #Advanced setting for identification | | |
| Relative abundance cut off | | 0 |

| | | |
|---|---|---|
| Top candidate report | False | |
| #Adduct ion setting | | |
| [M+H]+ | | |
| #Alignment parameters setting | | |
| Retention time tolerance | | 0.1 |
| MS1 tolerance | | 0.015 |
| Retention time factor | | 0.5 |
| MS1 factor | | 0.5 |
| Peak count filter | | 75 |
| N% detected in at least one group | | 0 |
| Remove feature based on peak height fold-change | False | |
| Sample max / blank average | | 5 |
| Sample average / blank average | | 5 |
| Keep identified and annotated metabolites | True | |
| Keep removable features and assign the tag for checking | True | |
| Gap filling by compulsion | True | |
| #Tracking of isotope labels | | |
| Tracking of isotopic labels | FALSE | |
| #Ion mobility | | |
| Ion mobility data | FALSE | |

**Table S2.** Full list annotated lipids. RT indicates retention time in minutes, m/z indicates mass-to-charge ratio. Lipid nomenclature was kept as reported by MS-DIAL.

| Lipid | Class | Category | Adduct | RT | m/z |
|---|---|---|---|---|---|
| CAR 16:0 | CAR | Fatty acyls | [M+H]+ | 2.874 | 400.34119 |
| CAR 16:1 | CAR | Fatty acyls | [M+H]+ | 2.352 | 398.3259 |
| CAR 18:0 | CAR | Fatty acyls | [M+H]+ | 3.583 | 428.37402 |
| CAR 18:1 | CAR | Fatty acyls | [M+H]+ | 3.098 | 426.35651 |
| CE 18:1 | CE | Sterol lipids | [M+NH4]+ | 15.564 | 668.63318 |
| CE 18:2 | CE | Sterol lipids | [M+NH4]+ | 15.268 | 666.61816 |
| CE 18:3 | CE | Sterol lipids | [M+NH4]+ | 14.949 | 664.60156 |
| CE 20:4 | CE | Sterol lipids | [M+NH4]+ | 15.061 | 690.61951 |
| CE 22:5 | CE | Sterol lipids | [M+NH4]+ | 15.083 | 716.63367 |
| CE 22:6 | CE | Sterol lipids | [M+NH4]+ | 14.842 | 714.61835 |
| Cer 34:1;2O\|Cer 18:1;2O/16:0 | Cer | Sphingolipids | [M+H-H2O]+ | 7.323 | 520.50739 |
| Cer 40:1;2O\|Cer 18:1;2O/22:0 | Cer | Sphingolipids | [M+H-H2O]+ | 11.354 | 604.60071 |
| Cer 41:1;2O\|Cer 18:1;2O/23:0 | Cer | Sphingolipids | [M+CH3COO]- | 13.713 | 618.61993 |
| Cer 42:1;2O\|Cer 18:1;2O/24:0 | Cer | Sphingolipids | [M+H-H2O]+ | 13.099 | 632.63452 |
| Cer 42:2;2O\|Cer 18:1;2O/24:1 | Cer | Sphingolipids | [M+H-H2O]+ | 11.407 | 630.61908 |
| Cer 42:3;2O\|Cer 18:1;2O/24:2 | Cer | Sphingolipids | [M+H]+ | 10.149 | 646.61139 |
| Cer 43:1;2O\|Cer 18:1;2O/25:0 | Cer | Sphingolipids | [M+H-H2O]+ | 13.72 | 646.65045 |
| Cer 44:1;2O\|Cer 18:1;2O/26:0 | Cer | Sphingolipids | [M+H]+ | 14.161 | 678.67511 |
| CoQ9 | Other | Other | [M+H]+ | 14.194 | 795.62512 |
| DG 32:1 | DG | Glycerolipids | [M+Na]+ | 8.514 | 589.48102 |
| DG 32:3 | DG | Glycerolipids | [M+Na]+ | 7.67 | 585.44458 |
| DG 34:0 | DG | Glycerolipids | [M+Na]+ | 10.888 | 619.52692 |
| DG 34:0 | DG | Glycerolipids | [M+Na]+ | 11.029 | 619.52704 |
| DG 34:1\|DG 16:0_18:1 | DG | Glycerolipids | [M+NH4]+ | 9.811 | 612.55798 |
| DG 34:2\|DG 16:0_18:2 | DG | Glycerolipids | [M+NH4]+ | 8.754 | 610.54199 |
| DG 34:3\|DG 16:1_18:2 | DG | Glycerolipids | [M+NH4]+ | 7.845 | 608.52588 |
| DG 36:2\|DG 18:1_18:1 | DG | Glycerolipids | [M+NH4]+ | 10.076 | 638.57361 |
| DG 36:3\|DG 18:1_18:2 | DG | Glycerolipids | [M+NH4]+ | 8.998 | 636.5578 |
| DG 36:4 | DG | Glycerolipids | [M+Na]+ | 8.503 | 639.49689 |
| DG 36:4\|DG 18:2_18:2 | DG | Glycerolipids | [M+NH4]+ | 8.055 | 634.54053 |
| DG 36:5\|DG 18:2_18:3 | DG | Glycerolipids | [M+NH4]+ | 7.467 | 632.52588 |
| DG 37:6 | DG | Glycerolipids | [M+Na]+ | 7.764 | 649.47607 |
| DG 37:7 | DG | Glycerolipids | [M+Na]+ | 12.363 | 647.45929 |
| DG 37:7 | DG | Glycerolipids | [M+Na]+ | 5.359 | 647.46216 |
| DG 38:2 | DG | Glycerolipids | [M+Na]+ | 15.268 | 671.57373 |
| DG 38:4 | DG | Glycerolipids | [M+Na]+ | 9.717 | 667.52722 |
| DG 38:5\|DG 18:1_20:4 | DG | Glycerolipids | [M+NH4]+ | 8.774 | 660.55737 |
| DG 38:6 | DG | Glycerolipids | [M+Na]+ | 7.861 | 663.49683 |
| DG 40:2 | DG | Glycerolipids | [M+Na]+ | 13.247 | 699.59332 |
| DG 40:4 | DG | Glycerolipids | [M+Na]+ | 15.062 | 695.57367 |
| DG 40:5 | DG | Glycerolipids | [M+Na]+ | 9.735 | 693.54358 |
| DG 40:5 | DG | Glycerolipids | [M+Na]+ | 14.708 | 693.55902 |

| | | | | | |
|---|---|---|---|---|---|
| DG 40:6 | DG | Glycerolipids | [M+Na]+ | 9.266 | 691.52759 |
| DG 40:8 | DG | Glycerolipids | [M+Na]+ | 11.449 | 687.49689 |
| DG 42:4 | DG | Glycerolipids | [M+Na]+ | 15.401 | 723.60431 |
| DG 42:5 | DG | Glycerolipids | [M+Na]+ | 15.156 | 721.58972 |
| DG 42:6 | DG | Glycerolipids | [M+Na]+ | 14.842 | 719.57404 |
| DG 43:6 | DG | Glycerolipids | [M+Na]+ | 14.333 | 733.57483 |
| DG 44:9 | DG | Glycerolipids | [M+Na]+ | 6.174 | 741.52887 |
| DG 49:6 | DG | Glycerolipids | [M+Na]+ | 15.461 | 817.66711 |
| DG 49:8 | DG | Glycerolipids | [M+Na]+ | 14.94 | 813.63739 |
| DG 51:10 | DG | Glycerolipids | [M+Na]+ | 14.669 | 837.63556 |
| DG 51:6 | DG | Glycerolipids | [M+Na]+ | 15.7 | 845.69781 |
| DG 51:7 | DG | Glycerolipids | [M+Na]+ | 15.497 | 843.68414 |
| DG 51:8 | DG | Glycerolipids | [M+Na]+ | 15.263 | 841.66589 |
| DG 51:9 | DG | Glycerolipids | [M+Na]+ | 14.995 | 839.65198 |
| DG 52:7 | DG | Glycerolipids | [M+Na]+ | 15.621 | 857.69855 |
| FA 14:0 | FA | Fatty acyls | [M-H]- | 3.475 | 227.2014 |
| FA 14:1 | FA | Fatty acyls | [M-H]- | 2.924 | 225.18555 |
| FA 15:0 | FA | Fatty acyls | [M-H]- | 3.849 | 241.2168 |
| FA 16:0 | FA | Fatty acyls | [M-H]- | 4.192 | 255.23264 |
| FA 16:1 | FA | Fatty acyls | [M-H]- | 3.693 | 253.21706 |
| FA 17:0 | FA | Fatty acyls | [M-H]- | 4.512 | 269.2486 |
| FA 17:1 | FA | Fatty acyls | [M-H]- | 4.039 | 267.23315 |
| FA 18:0 | FA | Fatty acyls | [M-H]- | 4.812 | 283.26382 |
| FA 18:1 | FA | Fatty acyls | [M-H]- | 4.364 | 281.24863 |
| FA 18:2 | FA | Fatty acyls | [M-H]- | 3.907 | 279.23309 |
| FA 18:3 | FA | Fatty acyls | [M-H]- | 3.469 | 277.21722 |
| FA 18:3 | FA | Fatty acyls | [M-H]- | 3.547 | 277.21722 |
| FA 19:1 | FA | Fatty acyls | [M-H]- | 4.653 | 295.26395 |
| FA 20:0 | FA | Fatty acyls | [M-H]- | 5.44 | 311.29605 |
| FA 20:1 | FA | Fatty acyls | [M-H]- | 4.912 | 309.27969 |
| FA 20:2 | FA | Fatty acyls | [M-H]- | 4.506 | 307.26425 |
| FA 20:3 | FA | Fatty acyls | [M-H]- | 4.28 | 305.24866 |
| FA 20:4 | FA | Fatty acyls | [M-H]- | 3.867 | 303.23257 |
| FA 20:4;O | FA | Fatty acyls | [M-H]- | 2.508 | 319.22797 |
| FA 20:5 | FA | Fatty acyls | [M-H]- | 3.44 | 301.21756 |
| FA 21:0 | FA | Fatty acyls | [M-H]- | 5.812 | 325.31146 |
| FA 22:0 | FA | Fatty acyls | [M-H]- | 6.248 | 339.3269 |
| FA 22:4 | FA | Fatty acyls | [M-H]- | 4.365 | 331.26465 |
| FA 22:5 | FA | Fatty acyls | [M-H]- | 3.995 | 329.24844 |
| FA 22:5 | FA | Fatty acyls | [M-H]- | 4.128 | 329.24893 |
| FA 22:6 | FA | Fatty acyls | [M-H]- | 3.743 | 327.23297 |
| FA 24:0 | FA | Fatty acyls | [M-H]- | 7.275 | 367.35779 |
| FA 24:1 | FA | Fatty acyls | [M-H]- | 6.332 | 365.3428 |
| FA 26:0 | FA | Fatty acyls | [M-H]- | 8.57 | 395.38977 |
| FA 26:1 | FA | Fatty acyls | [M-H]- | 7.388 | 393.37387 |
| FA 28:0 | FA | Fatty acyls | [M-H]- | 10.15 | 423.4213 |

| | | | | | |
|---|---|---|---|---|---|
| FA 28:7 | FA | Fatty acyls | [M-H]- | 5.865 | 409.31354 |
| FA 30:0 | FA | Fatty acyls | [M-H]- | 11.98 | 451.45239 |
| HexCer 34:1;2O | HexCer | Sphingolipids | [M+CH3COO]- | 7.061 | 758.57947 |
| HexCer 40:1;2O | HexCer | Sphingolipids | [M+CH3COO]- | 11.224 | 842.67322 |
| HexCer 41:1;2O | HexCer | Sphingolipids | [M+CH3COO]- | 12.147 | 856.68927 |
| HexCer 42:1;2O | HexCer | Sphingolipids | [M+CH3COO]- | 13.096 | 870.70569 |
| HexCer 42:2;2O | HexCer | Sphingolipids | [M+CH3COO]- | 11.278 | 868.6889 |
| LPC 14:0 | LPC | Glycerophospholipids | [M+H]+ | 1.801 | 468.3089 |
| LPC 14:0 | LPC | Glycerophospholipids | [M+H]+ | 1.955 | 468.30844 |
| LPC 15:0 | LPC | Glycerophospholipids | [M+H]+ | 2.353 | 482.32294 |
| LPC 16:0 | LPC | Glycerophospholipids | [M+H]+ | 2.579 | 496.34076 |
| LPC 16:0 | LPC | Glycerophospholipids | [M+H]+ | 2.738 | 496.34061 |
| LPC 16:1 | LPC | Glycerophospholipids | [M+H]+ | 2.204 | 494.32266 |
| LPC 17:0 | LPC | Glycerophospholipids | [M+H]+ | 3.113 | 510.35495 |
| LPC 17:1 | LPC | Glycerophospholipids | [M+H]+ | 2.59 | 508.34061 |
| LPC 18:0 | LPC | Glycerophospholipids | [M+H]+ | 3.325 | 524.36993 |
| LPC 18:0 | LPC | Glycerophospholipids | [M+H]+ | 3.471 | 524.36969 |
| LPC 18:1 | LPC | Glycerophospholipids | [M+H]+ | 2.816 | 522.35394 |
| LPC 18:1 | LPC | Glycerophospholipids | [M+H]+ | 2.96 | 522.35406 |
| LPC 18:2 | LPC | Glycerophospholipids | [M+H]+ | 2.316 | 520.33856 |
| LPC 18:2 | LPC | Glycerophospholipids | [M+H]+ | 2.462 | 520.33856 |
| LPC 18:3 | LPC | Glycerophospholipids | [M+H]+ | 2.114 | 518.32361 |
| LPC 19:0 | LPC | Glycerophospholipids | [M+H]+ | 3.802 | 538.38593 |
| LPC 20:0 | LPC | Glycerophospholipids | [M+H]+ | 4.12 | 552.40149 |
| LPC 20:1 | LPC | Glycerophospholipids | [M+H]+ | 3.607 | 550.38739 |
| LPC 20:2 | LPC | Glycerophospholipids | [M+H]+ | 3.142 | 548.37201 |
| LPC 20:3 | LPC | Glycerophospholipids | [M+H]+ | 2.753 | 546.35583 |
| LPC 20:4 | LPC | Glycerophospholipids | [M+H]+ | 2.357 | 544.33862 |
| LPC 20:4 | LPC | Glycerophospholipids | [M+H]+ | 2.475 | 544.33856 |
| LPC 22:0 | LPC | Glycerophospholipids | [M+H]+ | 4.705 | 580.43445 |
| LPC 22:1 | LPC | Glycerophospholipids | [M+H]+ | 4.212 | 578.41687 |
| LPC 22:4 | LPC | Glycerophospholipids | [M+H]+ | 3.043 | 572.37189 |
| LPC 22:5 | LPC | Glycerophospholipids | [M+H]+ | 2.624 | 570.35437 |
| LPC 22:5 | LPC | Glycerophospholipids | [M+H]+ | 2.842 | 570.35437 |
| LPC 22:6 | LPC | Glycerophospholipids | [M+H]+ | 2.286 | 568.34088 |
| LPC 22:6 | LPC | Glycerophospholipids | [M+H]+ | 2.408 | 568.34094 |
| LPC 24:0 | LPC | Glycerophospholipids | [M+H]+ | 5.211 | 608.46533 |
| LPC 24:1 | LPC | Glycerophospholipids | [M+H]+ | 4.733 | 606.44891 |
| LPE 16:0 | LPE | Glycerophospholipids | [M-H]- | 2.937 | 452.27856 |
| LPE 18:0 | LPE | Glycerophospholipids | [M-H]- | 4.184 | 480.30939 |
| LPE 18:1 | LPE | Glycerophospholipids | [M-H]- | 3.601 | 478.29391 |
| LPE 18:2 | LPE | Glycerophospholipids | [M-H]- | 2.678 | 476.2789 |
| PC 30:0\|PC 14:0_16:0 | PC | Glycerophospholipids | [M+CH3COO]- | 6.842 | 764.54523 |
| PC 31:0\|PC 15:0_16:0 | PC | Glycerophospholipids | [M+CH3COO]- | 7.33 | 778.5614 |
| PC 32:0 | PC | Glycerophospholipids | [M+H]+ | 6.361 | 734.56897 |
| PC 32:0\|PC 16:0_16:0 | PC | Glycerophospholipids | [M+CH3COO]- | 7.863 | 792.57556 |

| | | | | | |
|---|---|---|---|---|---|
| PC 32:1\|PC 16:0_16:1 | PC | Glycerophospholipids | [M+CH3COO]- | 7.071 | 790.56122 |
| PC 32:2\|PC 14:0_18:2 | PC | Glycerophospholipids | [M+CH3COO]- | 6.374 | 788.54724 |
| PC 33:0 | PC | Glycerophospholipids | [M+H]+ | 7.623 | 748.58331 |
| PC 33:1\|PC 15:0_18:1 | PC | Glycerophospholipids | [M+CH3COO]- | 7.578 | 804.57672 |
| PC 33:2\|PC 15:0_18:2 | PC | Glycerophospholipids | [M+CH3COO]- | 6.802 | 802.56323 |
| PC 34:0\|PC 16:0_18:0 | PC | Glycerophospholipids | [M+CH3COO]- | 9.147 | 820.60693 |
| PC 34:1\|PC 16:0_18:1 | PC | Glycerophospholipids | [M+CH3COO]- | 8.147 | 818.59143 |
| PC 34:2\|PC 16:0_18:2 | PC | Glycerophospholipids | [M+CH3COO]- | 7.288 | 816.57648 |
| PC 34:3 | PC | Glycerophospholipids | [M+H]+ | 6.295 | 756.5542 |
| PC 34:3\|PC 16:1_18:2 | PC | Glycerophospholipids | [M+CH3COO]- | 6.549 | 814.56073 |
| PC 34:4\|PC 14:0_20:4 | PC | Glycerophospholipids | [M+CH3COO]- | 6.34 | 812.54675 |
| PC 35:0 | PC | Glycerophospholipids | [M+H]+ | 8.725 | 776.61792 |
| PC 35:1 | PC | Glycerophospholipids | [M+H]+ | 7.87 | 774.59985 |
| PC 35:2\|PC 17:0_18:2 | PC | Glycerophospholipids | [M+CH3COO]- | 7.832 | 830.59357 |
| PC 35:3 | PC | Glycerophospholipids | [M+H]+ | 6.441 | 770.56824 |
| PC 35:4 | PC | Glycerophospholipids | [M+H]+ | 6.234 | 768.55249 |
| PC 36:0\|PC 18:0_18:0 | PC | Glycerophospholipids | [M+CH3COO]- | 10.636 | 848.6402 |
| PC 36:1\|PC 18:0_18:1 | PC | Glycerophospholipids | [M+CH3COO]- | 9.482 | 846.62549 |
| PC 36:2\|PC 18:0_18:2 | PC | Glycerophospholipids | [M+CH3COO]- | 8.451 | 844.60828 |
| PC 36:2\|PC 18:1_18:1 | PC | Glycerophospholipids | [M+H]+ | 7.589 | 786.59766 |
| PC 36:3\|PC 16:0_20:3 | PC | Glycerophospholipids | [M+CH3COO]- | 7.596 | 842.59229 |
| PC 36:3\|PC 16:0_20:3 | PC | Glycerophospholipids | [M+CH3COO]- | 8.03 | 842.59235 |
| PC 36:3\|PC 16:0_20:3 | PC | Glycerophospholipids | [M+CH3COO]- | 7.793 | 842.59265 |
| PC 36:3\|PC 18:1_18:2 | PC | Glycerophospholipids | [M+CH3COO]- | 7.497 | 842.59235 |
| PC 36:4 | PC | Glycerophospholipids | [M+H]+ | 6.618 | 782.56964 |
| PC 36:4 | PC | Glycerophospholipids | [M+H]+ | 6.22 | 782.56964 |
| PC 36:4\|PC 16:0_20:4 | PC | Glycerophospholipids | [M+CH3COO]- | 7.241 | 840.57556 |
| PC 36:4\|PC 18:2_18:2 | PC | Glycerophospholipids | [M+CH3COO]- | 6.744 | 840.57776 |
| PC 36:5\|PC 16:1_20:4 | PC | Glycerophospholipids | [M+CH3COO]- | 6.506 | 838.56226 |
| PC 36:6\|PC 14:0_22:6 | PC | Glycerophospholipids | [M+CH3COO]- | 6.127 | 836.54596 |
| PC 37:1 | PC | Glycerophospholipids | [M+H]+ | 9.057 | 802.63239 |
| PC 37:2\|PC 19:0_18:2 | PC | Glycerophospholipids | [M+CH3COO]- | 9.125 | 858.62518 |
| PC 37:2\|PC 19:0_18:2 | PC | Glycerophospholipids | [M+CH3COO]- | 8.969 | 858.62585 |
| PC 37:4 | PC | Glycerophospholipids | [M+CH3COO]- | 8.387 | 854.59253 |
| PC 37:4\|PC 17:0_20:4 | PC | Glycerophospholipids | [M+CH3COO]- | 7.785 | 854.59308 |
| PC 37:5 | PC | Glycerophospholipids | [M+H]+ | 6.529 | 794.56714 |
| PC 37:6\|PC 15:0_22:6 | PC | Glycerophospholipids | [M+CH3COO]- | 6.522 | 850.5603 |
| PC 38:1 | PC | Glycerophospholipids | [M+H]+ | 9.525 | 816.64935 |
| PC 38:2\|PC 18:0_20:2 | PC | Glycerophospholipids | [M+CH3COO]- | 9.63 | 872.64038 |
| PC 38:2\|PC 20:0_18:2 | PC | Glycerophospholipids | [M+CH3COO]- | 9.869 | 872.64026 |
| PC 38:3\|PC 18:0_20:3 | PC | Glycerophospholipids | [M+CH3COO]- | 8.82 | 870.625 |
| PC 38:3\|PC 18:0_20:3 | PC | Glycerophospholipids | [M+CH3COO]- | 9.07 | 870.62732 |
| PC 38:4\|PC 16:0_22:4 | PC | Glycerophospholipids | [M+CH3COO]- | 7.99 | 868.60889 |
| PC 38:4\|PC 18:0_20:4 | PC | Glycerophospholipids | [M+CH3COO]- | 8.389 | 868.60724 |
| PC 38:5\|PC 16:0_22:5 | PC | Glycerophospholipids | [M+CH3COO]- | 7.231 | 866.59143 |
| PC 38:5\|PC 16:0_22:5 | PC | Glycerophospholipids | [M+CH3COO]- | 7.663 | 866.59155 |

| | | | | | |
|---|---|---|---|---|---|
| PC 38:5\|PC 18:1_20:4 | PC | Glycerophospholipids | [M+CH3COO]- | 7.432 | 866.59174 |
| PC 38:6\|PC 16:0_22:6 | PC | Glycerophospholipids | [M+CH3COO]- | 6.965 | 864.57776 |
| PC 38:6\|PC 18:2_20:4 | PC | Glycerophospholipids | [M+CH3COO]- | 6.698 | 864.57715 |
| PC 38:7 | PC | Glycerophospholipids | [M+H]+ | 5.847 | 804.54456 |
| PC 39:5\|PC 17:0_22:5 | PC | Glycerophospholipids | [M+CH3COO]- | 8.238 | 880.60712 |
| PC 39:6\|PC 17:0_22:6 | PC | Glycerophospholipids | [M+CH3COO]- | 7.464 | 878.59351 |
| PC 39:7 | PC | Glycerophospholipids | [M+H]+ | 7.057 | 818.56659 |
| PC 40:2 | PC | Glycerophospholipids | [M+H]+ | 10.146 | 842.66113 |
| PC 40:3 | PC | Glycerophospholipids | [M+H]+ | 9.135 | 840.64905 |
| PC 40:4 | PC | Glycerophospholipids | [M+H]+ | 8.663 | 838.63239 |
| PC 40:4\|PC 18:0_22:4 | PC | Glycerophospholipids | [M+CH3COO]- | 9.282 | 896.64136 |
| PC 40:4\|PC 20:0_20:4 | PC | Glycerophospholipids | [M+CH3COO]- | 9.815 | 896.63989 |
| PC 40:5 | PC | Glycerophospholipids | [M+H]+ | 7.63 | 836.61499 |
| PC 40:5\|PC 18:0_22:5 | PC | Glycerophospholipids | [M+CH3COO]- | 8.901 | 894.62378 |
| PC 40:5\|PC 18:0_22:5 | PC | Glycerophospholipids | [M+CH3COO]- | 8.364 | 894.62335 |
| PC 40:6\|PC 18:0_22:6 | PC | Glycerophospholipids | [M+CH3COO]- | 8.036 | 892.60651 |
| PC 40:6\|PC 20:2_20:4 | PC | Glycerophospholipids | [M+CH3COO]- | 7.603 | 892.60919 |
| PC 40:7\|PC 18:1_22:6 | PC | Glycerophospholipids | [M+CH3COO]- | 7.136 | 890.59186 |
| PC 40:8\|PC 18:2_22:6 | PC | Glycerophospholipids | [M+CH3COO]- | 6.463 | 888.57587 |
| PC 40:8\|PC 20:4_20:4 | PC | Glycerophospholipids | [M+CH3COO]- | 6.602 | 888.57648 |
| PC 40:9 | PC | Glycerophospholipids | [M+H]+ | 5.663 | 828.55707 |
| PC 41:5 | PC | Glycerophospholipids | [M+H]+ | 8.45 | 850.6286 |
| PC 42:10\|PC 20:4_22:6 | PC | Glycerophospholipids | [M+CH3COO]- | 6.384 | 912.57672 |
| PC 42:5 | PC | Glycerophospholipids | [M+H]+ | 8.745 | 864.64746 |
| PC 42:6\|PC 18:0_24:6 | PC | Glycerophospholipids | [M+CH3COO]- | 8.822 | 920.63959 |
| PC 42:8 | PC | Glycerophospholipids | [M+H]+ | 6.635 | 858.59662 |
| PC 42:9\|PC 20:4_22:5 | PC | Glycerophospholipids | [M+CH3COO]- | 6.99 | 914.59381 |
| PC O-32:0\|PC O-16:0_16:0 | PC-O | Glycerophospholipids | [M+CH3COO]- | 8.57 | 778.59814 |
| PC O-32:1 | PC-O | Glycerophospholipids | [M+H]+ | 7.666 | 718.57568 |
| PC O-32:1 | PC-O | Glycerophospholipids | [M+H]+ | 6.979 | 718.57562 |
| PC O-34:0 | PC-O | Glycerophospholipids | [M+H]+ | 8.904 | 748.62408 |
| PC O-34:1\|PC O-18:1_16:0 | PC-O | Glycerophospholipids | [M+CH3COO]- | 8.8 | 804.61407 |
| PC O-34:2 | PC-O | Glycerophospholipids | [M+H]+ | 7.17 | 744.58868 |
| PC O-34:3\|PC O-16:1_18:2 | PC-O | Glycerophospholipids | [M+CH3COO]- | 7.838 | 800.58124 |
| PC O-36:3 | PC-O | Glycerophospholipids | [M+H]+ | 7.317 | 770.60413 |
| PC O-36:3 | PC-O | Glycerophospholipids | [M+H]+ | 7.448 | 770.60333 |
| PC O-36:4\|PC O-16:0_20:4 | PC-O | Glycerophospholipids | [M+CH3COO]- | 7.85 | 826.59863 |
| PC O-36:5\|PC O-16:1_20:4 | PC-O | Glycerophospholipids | [M+CH3COO]- | 7.751 | 824.58234 |
| PC O-38:4\|PC O-16:0_22:4 | PC-O | Glycerophospholipids | [M+CH3COO]- | 8.687 | 854.63074 |
| PC O-38:4\|PC O-18:0_20:4 | PC-O | Glycerophospholipids | [M+CH3COO]- | 9.153 | 854.62836 |
| PC O-38:5\|PC O-16:1_22:4 | PC-O | Glycerophospholipids | [M+CH3COO]- | 8.343 | 852.61426 |
| PC O-38:5\|PC O-18:1_20:4 | PC-O | Glycerophospholipids | [M+CH3COO]- | 8.051 | 852.61243 |
| PC O-38:6\|PC O-16:0_22:6 | PC-O | Glycerophospholipids | [M+CH3COO]- | 7.511 | 850.59717 |
| PC O-38:6\|PC O-18:2_20:4 | PC-O | Glycerophospholipids | [M+CH3COO]- | 7.948 | 850.5976 |
| PC O-38:7\|PC O-16:1_22:6 | PC-O | Glycerophospholipids | [M+CH3COO]- | 7.426 | 848.5827 |
| PC O-39:10 | PC-O | Glycerophospholipids | [M+H]+ | 7.358 | 798.53906 |

| | | | | | |
|---|---|---|---|---|---|
| PC O-40:4\|PC O-20:0_20:4 | PC-O | Glycerophospholipids | [M+CH3COO]- | 10.725 | 882.66107 |
| PC O-40:7\|PC O-18:1_22:6 | PC-O | Glycerophospholipids | [M+CH3COO]- | 7.692 | 876.61249 |
| PC O-40:8 | PC-O | Glycerophospholipids | [M+H]+ | 7.266 | 816.59113 |
| PC O-41:10 | PC-O | Glycerophospholipids | [M+H]+ | 8.438 | 826.57202 |
| PC O-41:11 | PC-O | Glycerophospholipids | [M+H]+ | 7.58 | 824.55493 |
| PE 34:2\|PE 16:0_18:2 | PE | Glycerophospholipids | [M-H]- | 7.468 | 714.5105 |
| PE 36:2\|PE 18:0_18:2 | PE | Glycerophospholipids | [M-H]- | 8.669 | 742.53906 |
| PE 36:4\|PE 16:0_20:4 | PE | Glycerophospholipids | [M-H]- | 7.412 | 738.51044 |
| PE 38:4\|PE 18:0_20:4 | PE | Glycerophospholipids | [M-H]- | 8.601 | 766.53998 |
| PE 38:5\|PE 18:1_20:4 | PE | Glycerophospholipids | [M-H]- | 7.658 | 764.52545 |
| PE 38:6\|PE 16:0_22:6 | PE | Glycerophospholipids | [M-H]- | 7.12 | 762.51025 |
| PE 40:6\|PE 18:0_22:6 | PE | Glycerophospholipids | [M-H]- | 6.968 | 790.53973 |
| PE O-36:5\|PE O-16:1_20:4 | PE-O | Glycerophospholipids | [M-H]- | 7.988 | 722.51453 |
| PE O-38:5\|PE O-18:1_20:4 | PE-O | Glycerophospholipids | [M-H]- | 9.304 | 750.5459 |
| PE O-38:6\|PE O-18:2_20:4 | PE-O | Glycerophospholipids | [M-H]- | 8.192 | 748.5293 |
| PE P-36:4\|PE P-16:0_20:4 | PE-P | Glycerophospholipids | [M+H]+ | 7.245 | 724.52771 |
| PE P-38:5\|PE P-18:1_20:4 | PE-P | Glycerophospholipids | [M+H]+ | 7.403 | 750.54468 |
| PI 36:4 | PI | Glycerophospholipids | [M-H]- | 6.046 | 857.52344 |
| PI 38:4\|PI 18:0_20:4 | PI | Glycerophospholipids | [M-H]- | 6.862 | 885.55011 |
| SE 28:1/20:4 | Other | Other | [M+NH4]+ | 15.236 | 704.63147 |
| SE 29:1/20:4 | Other | Other | [M+NH4]+ | 15.402 | 718.64874 |
| SE 29:2/20:4 | Other | Other | [M+NH4]+ | 15.158 | 716.63367 |
| SM 32:1;2O | SM | Sphingolipids | [M+H]+ | 5.516 | 675.54181 |
| SM 33:1;2O | SM | Sphingolipids | [M+H]+ | 5.834 | 689.55804 |
| SM 34:0;2O | SM | Sphingolipids | [M+H]+ | 6.41 | 705.591 |
| SM 34:1;2O | SM | Sphingolipids | [M+H]+ | 6.172 | 703.57684 |
| SM 34:1;3O | SM | Sphingolipids | [M+H]+ | 5.939 | 719.5686 |
| SM 34:2;2O | SM | Sphingolipids | [M+H]+ | 5.639 | 701.55902 |
| SM 34:2;3O | SM | Sphingolipids | [M+H]+ | 5.454 | 717.55292 |
| SM 35:1;2O | SM | Sphingolipids | [M+H]+ | 6.568 | 717.59039 |
| SM 36:1;2O | SM | Sphingolipids | [M+H]+ | 6.996 | 731.60822 |
| SM 36:2;2O | SM | Sphingolipids | [M+H]+ | 6.343 | 729.58997 |
| SM 38:1;2O | SM | Sphingolipids | [M+H]+ | 8.051 | 759.63727 |
| SM 39:1;2O | SM | Sphingolipids | [M+H]+ | 8.682 | 773.65027 |
| SM 40:1;2O | SM | Sphingolipids | [M+H]+ | 9.336 | 787.66608 |
| SM 40:2;2O | SM | Sphingolipids | [M+H]+ | 8.332 | 785.65222 |
| SM 40:2;2O | SM | Sphingolipids | [M+H]+ | 8.141 | 785.6521 |
| SM 41:1;2O | SM | Sphingolipids | [M+H]+ | 10.072 | 801.68402 |
| SM 41:2;2O | SM | Sphingolipids | [M+H]+ | 8.989 | 799.66962 |
| SM 41:2;2O | SM | Sphingolipids | [M+H]+ | 8.739 | 799.66931 |
| SM 42:1;2O | SM | Sphingolipids | [M+H]+ | 10.831 | 815.7002 |
| SM 42:2;2O | SM | Sphingolipids | [M+H]+ | 9.346 | 813.68555 |
| SM 42:2;2O | SM | Sphingolipids | [M+H]+ | 9.686 | 813.68085 |
| SM 42:3;2O | SM | Sphingolipids | [M+H]+ | 8.339 | 811.66962 |
| SM 43:1;2O | SM | Sphingolipids | [M+H]+ | 11.494 | 829.71619 |
| SM 43:1;2O | SM | Sphingolipids | [M+H]+ | 11.672 | 829.71478 |

| | | | | | |
|---|---|---|---|---|---|
| SM 43:2;2O | SM | Sphingolipids | [M+H]+ | 10.244 | 827.70178 |
| ST 24:1;O4 | ST | Sterol lipids | [M-H]- | 1.197 | 391.28586 |
| ST 24:1;O4 | ST | Sterol lipids | [M-H]- | 1.825 | 391.28589 |
| ST 24:1;O4 | ST | Sterol lipids | [M-H]- | 1.972 | 391.28595 |
| ST 27:1;O | ST | Sterol lipids | [M+H-H2O]+ | 5.498 | 369.3512 |
| ST 27:1;O | ST | Sterol lipids | [M+H-H2O]+ | 3.886 | 369.35114 |
| ST 27:1;O;S | ST | Sterol lipids | [M-H]- | 3.996 | 465.30414 |
| ST 28:1;O;S | ST | Sterol lipids | [M-H]- | 4.178 | 479.3201 |
| TG 42:1\|TG 12:0_12:0_18:1 | TG | Glycerolipids | [M+NH4]+ | 14.468 | 738.65985 |
| TG 44:0\|TG 14:0_14:0_16:0 | TG | Glycerolipids | [M+NH4]+ | 15.176 | 768.70807 |
| TG 44:1\|TG 12:0_16:0_16:1 | TG | Glycerolipids | [M+NH4]+ | 15.061 | 766.69147 |
| TG 44:1\|TG 14:0_16:0_14:1 | TG | Glycerolipids | [M+NH4]+ | 14.911 | 766.69116 |
| TG 45:0\|TG 14:0_15:0_16:0 | TG | Glycerolipids | [M+NH4]+ | 15.325 | 782.71918 |
| TG 45:1\|TG 14:0_15:0_16:1 | TG | Glycerolipids | [M+NH4]+ | 15.083 | 780.70911 |
| TG 46:0\|TG 14:0_16:0_16:0 | TG | Glycerolipids | [M+NH4]+ | 15.467 | 796.73883 |
| TG 46:1\|TG 14:0_16:0_16:1 | TG | Glycerolipids | [M+NH4]+ | 15.248 | 794.72321 |
| TG 46:2\|TG 12:0_16:0_18:2 | TG | Glycerolipids | [M+NH4]+ | 15.032 | 792.70605 |
| TG 46:3\|TG 12:0_16:1_18:2 | TG | Glycerolipids | [M+NH4]+ | 14.595 | 790.69354 |
| TG 47:0\|TG 15:0_16:0_16:0 | TG | Glycerolipids | [M+NH4]+ | 15.583 | 810.75439 |
| TG 47:1\|TG 15:0_16:0_16:1 | TG | Glycerolipids | [M+NH4]+ | 15.39 | 808.73889 |
| TG 48:0\|TG 16:0_16:0_16:0 | TG | Glycerolipids | [M+NH4]+ | 15.709 | 824.77197 |
| TG 48:1\|TG 14:0_16:0_18:1 | TG | Glycerolipids | [M+NH4]+ | 15.502 | 822.75281 |
| TG 48:2\|TG 14:0_16:0_18:2 | TG | Glycerolipids | [M+NH4]+ | 15.265 | 820.73914 |
| TG 48:4\|TG 12:0_18:2_18:2 | TG | Glycerolipids | [M+NH4]+ | 14.681 | 816.70428 |
| TG 49:0\|TG 15:0_16:0_18:0 | TG | Glycerolipids | [M+NH4]+ | 15.815 | 838.7876 |
| TG 49:1\|TG 15:0_16:0_18:1 | TG | Glycerolipids | [M+NH4]+ | 15.62 | 836.76892 |
| TG 49:2\|TG 16:0_16:1_17:1 | TG | Glycerolipids | [M+NH4]+ | 15.406 | 834.75317 |
| TG 49:3\|TG 15:0_16:1_18:2 | TG | Glycerolipids | [M+NH4]+ | 15.145 | 832.73785 |
| TG 50:0\|TG 16:0_16:0_18:0 | TG | Glycerolipids | [M+NH4]+ | 15.923 | 852.80359 |
| TG 50:1\|TG 16:0_16:0_18:1 | TG | Glycerolipids | [M+NH4]+ | 15.731 | 850.78589 |
| TG 50:2\|TG 16:0_16:1_18:1 | TG | Glycerolipids | [M+NH4]+ | 15.528 | 848.76862 |
| TG 50:3\|TG 16:0_16:1_18:2 | TG | Glycerolipids | [M+NH4]+ | 15.295 | 846.75311 |
| TG 50:4\|TG 16:1_16:1_18:2 | TG | Glycerolipids | [M+NH4]+ | 15.054 | 844.74091 |
| TG 50:5\|TG 16:0_16:1_18:4 | TG | Glycerolipids | [M+NH4]+ | 14.888 | 842.72449 |
| TG 51:0\|TG 16:0_17:0_18:0 | TG | Glycerolipids | [M+NH4]+ | 16.021 | 866.81622 |
| TG 51:1\|TG 16:0_17:0_18:1 | TG | Glycerolipids | [M+NH4]+ | 15.835 | 864.79895 |
| TG 51:2\|TG 16:0_17:1_18:1 | TG | Glycerolipids | [M+NH4]+ | 15.643 | 862.78784 |
| TG 51:3\|TG 15:0_18:1_18:2 | TG | Glycerolipids | [M+NH4]+ | 15.431 | 860.77222 |
| TG 51:4\|TG 15:0_18:2_18:2 | TG | Glycerolipids | [M+NH4]+ | 15.272 | 858.75171 |
| TG 52:0\|TG 16:0_18:0_18:0 | TG | Glycerolipids | [M+NH4]+ | 16.12 | 880.8299 |
| TG 52:1\|TG 16:0_18:0_18:1 | TG | Glycerolipids | [M+NH4]+ | 15.938 | 878.815 |
| TG 52:2\|TG 16:0_18:1_18:1 | TG | Glycerolipids | [M+NH4]+ | 15.751 | 876.80127 |
| TG 52:3\|TG 16:0_18:1_18:2 | TG | Glycerolipids | [M+NH4]+ | 15.547 | 874.7865 |
| TG 52:4\|TG 16:0_18:2_18:2 | TG | Glycerolipids | [M+NH4]+ | 15.322 | 872.7713 |
| TG 52:5\|TG 16:0_18:2_18:3 | TG | Glycerolipids | [M+NH4]+ | 15.063 | 870.75128 |
| TG 52:6\|TG 16:1_18:2_18:3 | TG | Glycerolipids | [M+NH4]+ | 14.934 | 868.73798 |

| | | | | | |
|---|---|---|---|---|---|
| TG 53:1\|TG 17:0_18:0_18:1 | TG | Glycerolipids | [M+NH4]+ | 16.035 | 892.83533 |
| TG 53:2\|TG 17:0_18:1_18:1 | TG | Glycerolipids | [M+NH4]+ | 15.851 | 890.81409 |
| TG 53:3\|TG 17:0_18:1_18:2 | TG | Glycerolipids | [M+NH4]+ | 15.661 | 888.79974 |
| TG 53:4\|TG 17:1_18:1_18:2 | TG | Glycerolipids | [M+NH4]+ | 15.547 | 886.78619 |
| TG 53:5\|TG 16:0_18:2_19:3 | TG | Glycerolipids | [M+NH4]+ | 15.338 | 884.76849 |
| TG 53:6\|TG 15:0_18:2_20:4 | TG | Glycerolipids | [M+NH4]+ | 15.09 | 882.75458 |
| TG 54:0\|TG 16:0_18:0_20:0 | TG | Glycerolipids | [M+NH4]+ | 16.32 | 908.86292 |
| TG 54:2\|TG 18:0_18:1_18:1 | TG | Glycerolipids | [M+NH4]+ | 15.951 | 904.83105 |
| TG 54:3\|TG 18:0_18:1_18:2 | TG | Glycerolipids | [M+NH4]+ | 15.768 | 902.81812 |
| TG 54:4\|TG 16:0_18:1_20:3 | TG | Glycerolipids | [M+NH4]+ | 15.569 | 900.80103 |
| TG 54:4\|TG 18:1_18:1_18:2 | TG | Glycerolipids | [M+NH4]+ | 15.659 | 900.80206 |
| TG 54:5\|TG 16:0_18:1_20:4 | TG | Glycerolipids | [M+NH4]+ | 15.359 | 898.78558 |
| TG 54:6\|TG 16:0_18:2_20:4 | TG | Glycerolipids | [M+NH4]+ | 15.233 | 896.77039 |
| TG 54:7\|TG 16:0_18:2_20:5 | TG | Glycerolipids | [M+NH4]+ | 15.053 | 894.75494 |
| TG 54:7\|TG 18:2_18:2_18:3 | TG | Glycerolipids | [M+NH4]+ | 14.987 | 894.75415 |
| TG 55:0\|TG 15:0_16:0_24:0 | TG | Glycerolipids | [M+NH4]+ | 16.433 | 922.88165 |
| TG 55:2\|TG 17:0_18:1_20:1 | TG | Glycerolipids | [M+NH4]+ | 16.045 | 918.84735 |
| TG 55:3\|TG 19:0_18:1_18:2 | TG | Glycerolipids | [M+NH4]+ | 15.868 | 916.83527 |
| TG 55:4\|TG 17:0_18:1_20:3 | TG | Glycerolipids | [M+NH4]+ | 15.691 | 914.8222 |
| TG 55:5\|TG 17:0_18:1_20:4 | TG | Glycerolipids | [M+NH4]+ | 15.581 | 912.79901 |
| TG 55:6\|TG 16:0_17:0_22:6 | TG | Glycerolipids | [M+NH4]+ | 15.365 | 910.78284 |
| TG 56:0\|TG 16:0_16:0_24:0 | TG | Glycerolipids | [M+NH4]+ | 16.529 | 936.8949 |
| TG 56:1\|TG 16:0_18:0_22:1 | TG | Glycerolipids | [M+NH4]+ | 16.327 | 934.87781 |
| TG 56:2\|TG 16:0_22:0_18:2 | TG | Glycerolipids | [M+NH4]+ | 16.127 | 932.86102 |
| TG 56:3\|TG 16:0_22:1_18:2 | TG | Glycerolipids | [M+NH4]+ | 15.938 | 930.85071 |
| TG 56:4\|TG 16:0_18:1_22:3 | TG | Glycerolipids | [M+NH4]+ | 15.78 | 928.83405 |
| TG 56:5\|TG 16:0_18:1_22:4 | TG | Glycerolipids | [M+NH4]+ | 15.684 | 926.81653 |
| TG 56:6\|TG 16:0_18:2_22:4 | TG | Glycerolipids | [M+NH4]+ | 15.533 | 924.79901 |
| TG 56:7\|TG 18:1_18:2_20:4 | TG | Glycerolipids | [M+NH4]+ | 15.284 | 922.78149 |
| TG 56:8\|TG 18:2_18:2_20:4 | TG | Glycerolipids | [M+NH4]+ | 15.098 | 920.77075 |
| TG 56:9\|TG 18:2_18:2_20:5 | TG | Glycerolipids | [M+NH4]+ | 14.821 | 918.75299 |
| TG 56:9\|TG 18:2_18:3_20:4 | TG | Glycerolipids | [M+NH4]+ | 14.899 | 918.7536 |
| TG 57:0\|TG 16:0_17:0_24:0 | TG | Glycerolipids | [M+NH4]+ | 16.635 | 950.90869 |
| TG 57:1\|TG 16:0_23:0_18:1 | TG | Glycerolipids | [M+NH4]+ | 16.446 | 948.89398 |
| TG 57:3\|TG 18:1_18:1_21:1 | TG | Glycerolipids | [M+NH4]+ | 16.064 | 944.86707 |
| TG 57:5\|TG 17:0_18:1_22:4 | TG | Glycerolipids | [M+NH4]+ | 15.781 | 940.83081 |
| TG 57:7\|TG 17:0_18:1_22:6 | TG | Glycerolipids | [M+NH4]+ | 15.469 | 936.80084 |
| TG 58:0\|TG 16:0_18:0_24:0 | TG | Glycerolipids | [M+NH4]+ | 16.742 | 964.92609 |
| TG 58:1\|TG 16:0_24:0_18:1 | TG | Glycerolipids | [M+NH4]+ | 16.526 | 962.91064 |
| TG 58:10\|TG 18:2_18:2_22:6 | TG | Glycerolipids | [M+NH4]+ | 14.94 | 944.76941 |
| TG 58:2\|TG 16:0_18:1_24:1 | TG | Glycerolipids | [M+NH4]+ | 16.335 | 960.89417 |
| TG 58:3\|TG 22:0_18:1_18:2 | TG | Glycerolipids | [M+NH4]+ | 16.149 | 958.87799 |
| TG 58:4\|TG 18:1_22:1_18:2 | TG | Glycerolipids | [M+NH4]+ | 15.951 | 956.85974 |
| TG 58:6\|TG 18:0_18:1_22:5 | TG | Glycerolipids | [M+NH4]+ | 15.75 | 952.8316 |
| TG 58:7\|TG 18:1_18:1_22:5 | TG | Glycerolipids | [M+NH4]+ | 15.564 | 950.81598 |
| TG 58:8\|TG 18:1_18:2_22:5 | TG | Glycerolipids | [M+NH4]+ | 15.399 | 948.79791 |

| | | | | | |
|---|---|---|---|---|---|
| TG 58:9\|TG 18:2_20:3_20:4 | TG | Glycerolipids | [M+NH4]+ | 15.203 | 946.78644 |
| TG 60:10\|TG 18:1_20:4_22:5 | TG | Glycerolipids | [M+NH4]+ | 15.291 | 972.80188 |
| TG 60:11\|TG 18:2_20:4_22:5 | TG | Glycerolipids | [M+NH4]+ | 15.056 | 970.7832 |
| TG 60:12\|TG 18:2_20:4_22:6 | TG | Glycerolipids | [M+NH4]+ | 14.846 | 968.76764 |
| TG 60:2\|TG 16:0_20:1_24:1 | TG | Glycerolipids | [M+NH4]+ | 16.527 | 988.92578 |
| TG 60:3\|TG 16:0_26:1_18:2 | TG | Glycerolipids | [M+NH4]+ | 16.335 | 986.90851 |
| TG 60:5\|TG 24:1_18:2_18:2 | TG | Glycerolipids | [M+NH4]+ | 16.042 | 982.87842 |
| TG 60:9\|TG 18:0_20:4_22:5 | TG | Glycerolipids | [M+NH4]+ | 15.456 | 974.81641 |
| TG 60:9\|TG 18:1_20:4_22:4 | TG | Glycerolipids | [M+NH4]+ | 15.219 | 974.81567 |
| TG 62:13\|TG 20:4_20:4_22:5 | TG | Glycerolipids | [M+NH4]+ | 14.954 | 994.78546 |
| TG 62:14\|TG 20:4_20:4_22:6 | TG | Glycerolipids | [M+NH4]+ | 14.701 | 992.77051 |
| TG 62:2\|TG 18:0_18:1_26:1 | TG | Glycerolipids | [M+NH4]+ | 16.727 | 1016.95728 |
| TG 62:4\|TG 18:1_26:1_18:2 | TG | Glycerolipids | [M+NH4]+ | 16.344 | 1012.9231 |

**Table S3.** List of *p*-values and log2 fold changes at the three time points for 377 lipids.

| Lipid | *p*-Value | | | log2 FoldChange | | | NI Associated |
|---|---|---|---|---|---|---|---|
| | Week 5 | Week 2 | Week 0 | Week 5 | Week 2 | Week 0 | |
| CAR 16:0 | 0.507 | 0.317 | 0.252 | 0.228 | -0.329 | -0.366 | No |
| CAR 16:1 | 0.522 | 0.661 | 0.455 | 0.719 | -0.281 | -0.550 | No |
| CAR 18:0 | 0.266 | 0.223 | 0.524 | 0.450 | -0.334 | -0.183 | No |
| CAR 18:1 | 0.827 | 0.193 | 0.455 | 0.085 | -0.442 | -0.262 | No |
| CE 18:1 | 0.292 | 0.515 | 0.135 | 0.394 | 0.196 | 0.408 | No |
| CE 18:2 | 0.572 | 0.674 | 0.578 | 0.192 | 0.100 | 0.133 | No |
| CE 18:3 | 0.355 | 0.117 | 0.875 | 0.563 | 0.584 | 0.085 | No |
| CE 20:4 | 0.016 | 0.862 | 0.016 | -0.441 | 0.027 | -0.268 | Yes |
| CE 22:5 | 0.044 | 0.452 | 0.249 | -0.900 | 0.254 | -0.252 | Yes |
| CE 22:6 | 0.609 | 0.404 | 0.714 | -0.201 | -0.252 | 0.084 | No |
| Cer 34:1;2O\|Cer 18:1;2O/16:0 | 0.278 | 0.492 | 0.521 | 0.546 | 0.328 | 0.206 | No |
| Cer 40:1;2O\|Cer 18:1;2O/22:0 | 0.079 | 0.282 | 0.190 | 0.776 | 0.405 | 0.548 | No |
| Cer 41:1;2O\|Cer 18:1;2O/23:0 | 0.007 | 0.467 | 0.185 | 0.644 | 0.134 | 0.339 | Yes |
| Cer 42:1;2O\|Cer 18:1;2O/24:0 | 0.057 | 0.170 | 0.274 | 0.581 | 0.337 | 0.307 | No |
| Cer 42:2;2O\|Cer 18:1;2O/24:1 | 0.096 | 0.322 | 0.408 | 0.878 | 0.365 | 0.282 | No |
| Cer 42:3;2O\|Cer 18:1;2O/24:2 | 0.293 | 0.507 | 0.850 | 0.846 | 0.513 | 0.094 | No |
| Cer 43:1;2O\|Cer 18:1;2O/25:0 | 0.185 | 0.345 | 0.930 | 0.950 | 0.693 | 0.035 | No |
| Cer 44:1;2O\|Cer 18:1;2O/26:0 | 0.254 | 0.671 | 0.087 | 0.587 | -0.161 | 0.930 | No |
| CoQ9 | 0.084 | 0.031 | 0.994 | 0.591 | 0.620 | 0.003 | No |
| DG 32:1 | 0.007 | 0.710 | 0.109 | 1.101 | 0.116 | -0.474 | Yes |
| DG 32:3 | 0.011 | 0.408 | 0.786 | -0.130 | -0.047 | -0.013 | No |
| DG 34:0 | 0.492 | 0.795 | 0.090 | 0.068 | -0.028 | -0.208 | No |
| DG 34:0 | 0.033 | 0.830 | 0.795 | 0.605 | 0.051 | -0.035 | Yes |
| DG 34:1\|DG 16:0_18:1 | 0.008 | 0.949 | 0.542 | 1.032 | 0.021 | -0.224 | Yes |
| DG 34:2\|DG 16:0_18:2 | 0.031 | 0.879 | 0.685 | 0.805 | 0.046 | -0.163 | Yes |
| DG 34:3\|DG 16:1_18:2 | 0.048 | 0.854 | 0.191 | 0.849 | 0.085 | -1.258 | Yes |
| DG 36:2\|DG 18:1_18:1 | 0.049 | 0.797 | 0.504 | 0.700 | -0.103 | 0.454 | Yes |
| DG 36:3\|DG 18:1_18:2 | 0.307 | 0.606 | 0.752 | 0.381 | -0.159 | -0.122 | No |
| DG 36:4 | 0.287 | 0.716 | 0.149 | 0.457 | -0.119 | -0.489 | No |
| DG 36:4\|DG 18:2_18:2 | 0.525 | 0.508 | 0.438 | 0.257 | -0.206 | -0.341 | No |
| DG 36:5\|DG 18:2_18:3 | 0.558 | 0.754 | 0.132 | -0.497 | -0.180 | -1.039 | No |
| DG 37:6 | 0.131 | 0.110 | 0.598 | -2.019 | -1.672 | 0.548 | No |
| DG 37:7 | 0.397 | 0.982 | 0.965 | -0.360 | -0.004 | -0.005 | No |
| DG 37:7 | 0.230 | 0.127 | 0.537 | -2.507 | -2.007 | -1.100 | No |
| DG 38:2 | 0.688 | 0.440 | 0.396 | -0.107 | 0.136 | 0.118 | No |
| DG 38:4 | 0.197 | 0.177 | 0.795 | 0.533 | 0.403 | -0.066 | No |
| DG 38:5\|DG 18:1_20:4 | 0.526 | 0.486 | 0.194 | -0.393 | -0.119 | -0.291 | No |
| DG 38:6 | 0.277 | 0.323 | 0.191 | -0.640 | -0.189 | -0.422 | No |
| DG 40:2 | 0.335 | 0.924 | 0.734 | 0.194 | 0.006 | 0.039 | No |
| DG 40:4 | 0.011 | 0.887 | 0.045 | -0.347 | 0.016 | -0.147 | Yes |
| DG 40:5 | 0.547 | 0.484 | 0.960 | 0.333 | 0.116 | -0.014 | No |
| DG 40:5 | 0.630 | 0.470 | 0.901 | -0.363 | 0.233 | 0.092 | No |
| DG 40:6 | 0.790 | 0.908 | 0.551 | -0.277 | -0.022 | -0.231 | No |

| | | | | | | | |
|---|---|---|---|---|---|---|---|
| DG 40:8 | 0.046 | 0.715 | 0.050 | -0.188 | 0.032 | -0.148 | No |
| DG 42:4 | 0.026 | 0.997 | 0.849 | -1.719 | -0.002 | -0.070 | Yes |
| DG 42:5 | 0.441 | 0.816 | 0.622 | 0.246 | 0.100 | -0.146 | No |
| DG 42:6 | 0.539 | 0.790 | 0.838 | -0.183 | -0.056 | 0.032 | No |
| DG 43:6 | 0.916 | 0.406 | 0.138 | 0.017 | 0.098 | 0.162 | No |
| DG 44:9 | 0.797 | 0.686 | 0.523 | -0.181 | 0.118 | 0.182 | No |
| DG 49:6 | 0.962 | 0.046 | 0.582 | 0.019 | 0.261 | -0.114 | No |
| DG 49:8 | 0.016 | 0.814 | 0.965 | 1.639 | 0.117 | -0.022 | Yes |
| DG 51:10 | 0.155 | 0.489 | 0.685 | 1.154 | 0.370 | -0.326 | No |
| DG 51:6 | 0.135 | 0.629 | 0.237 | 0.289 | -0.059 | -0.168 | No |
| DG 51:7 | 0.010 | 0.690 | 0.153 | 1.247 | 0.105 | -0.375 | Yes |
| DG 51:8 | 0.014 | 0.713 | 0.466 | 1.737 | 0.176 | -0.664 | Yes |
| DG 51:9 | 0.015 | 0.766 | 0.457 | 1.509 | 0.116 | -0.375 | Yes |
| DG 52:7 | 0.140 | 0.444 | 0.968 | 0.429 | 0.096 | -0.006 | No |
| FA 14:0 | 0.770 | 0.780 | 0.451 | -0.045 | 0.055 | -0.270 | No |
| FA 14:1 | 0.726 | 0.570 | 0.256 | -0.176 | 0.205 | -0.738 | No |
| FA 15:0 | 0.382 | 0.616 | 0.563 | 0.074 | -0.062 | 0.087 | No |
| FA 16:0 | 0.988 | 0.474 | 0.381 | 0.003 | -0.103 | -0.191 | No |
| FA 16:1 | 0.677 | 0.791 | 0.421 | -0.156 | 0.108 | -0.454 | No |
| FA 17:0 | 0.168 | 0.003 | 0.632 | 0.264 | -0.378 | -0.123 | No |
| FA 17:1 | 0.999 | 0.394 | 0.346 | 0.000 | -0.322 | -0.399 | No |
| FA 18:0 | 0.094 | 0.932 | 0.453 | -0.197 | -0.011 | -0.079 | No |
| FA 18:1 | 0.499 | 0.425 | 0.547 | -0.237 | -0.280 | -0.270 | No |
| FA 18:2 | 0.186 | 0.285 | 0.266 | -0.377 | -0.259 | -0.473 | No |
| FA 18:3 | 0.782 | 0.510 | 0.318 | -0.102 | -0.259 | -0.512 | No |
| FA 18:3 | 0.322 | 0.940 | 0.145 | -0.295 | -0.020 | -0.579 | No |
| FA 19:1 | 0.564 | 0.130 | 0.232 | -0.330 | -0.875 | -0.618 | No |
| FA 20:0 | 0.029 | 0.016 | 0.825 | 0.159 | -0.148 | -0.022 | No |
| FA 20:1 | 0.917 | 0.093 | 0.208 | 0.030 | -0.495 | -0.405 | No |
| FA 20:2 | 0.994 | 0.604 | 0.035 | -0.001 | -0.115 | -0.667 | No |
| FA 20:3 | 0.842 | 0.852 | 0.337 | 0.074 | 0.051 | -0.339 | No |
| FA 20:4 | 0.050 | 0.375 | 0.019 | -0.410 | -0.110 | -0.397 | Yes |
| FA 20:4;O | 0.617 | 0.384 | 0.780 | 0.800 | -0.815 | 0.240 | No |
| FA 20:5 | 0.957 | 0.349 | 0.010 | 0.018 | -0.272 | -1.002 | No |
| FA 21:0 | 0.058 | 0.718 | 0.129 | 0.225 | 0.092 | 0.195 | No |
| FA 22:0 | 0.163 | 0.700 | 0.732 | 0.165 | 0.040 | -0.039 | No |
| FA 22:4 | 0.089 | 0.876 | 0.018 | -0.655 | -0.032 | -0.701 | No |
| FA 22:5 | 0.303 | 0.252 | 0.014 | -0.490 | -0.260 | -0.750 | No |
| FA 22:5 | 0.724 | 0.557 | 0.057 | -0.169 | -0.100 | -0.574 | No |
| FA 22:6 | 0.238 | 0.278 | 0.148 | -0.380 | -0.230 | -0.292 | No |
| FA 24:0 | 0.303 | 0.777 | 0.775 | 0.112 | -0.027 | 0.031 | No |
| FA 24:1 | 0.237 | 0.076 | 0.813 | 0.384 | -0.352 | -0.069 | No |
| FA 26:0 | 0.726 | 0.587 | 0.928 | 0.041 | 0.060 | 0.011 | No |
| FA 26:1 | 0.194 | 0.240 | 0.787 | 0.680 | -0.759 | 0.146 | No |
| FA 28:0 | 0.879 | 0.553 | 0.994 | 0.020 | 0.090 | -0.001 | No |
| FA 28:7 | 0.143 | 0.624 | 0.608 | -0.303 | -0.132 | -0.170 | No |

| | | | | | | | |
|---|---|---|---|---|---|---|---|
| FA 30:0 | 0.786 | 0.549 | 0.728 | -0.034 | 0.095 | -0.059 | No |
| HexCer 34:1;2O | 0.225 | 0.633 | 0.373 | 0.490 | 0.146 | 0.316 | No |
| HexCer 40:1;2O | 0.109 | 0.118 | 0.123 | 0.424 | 0.357 | 0.462 | No |
| HexCer 41:1;2O | 0.686 | 0.800 | 0.143 | 0.146 | -0.077 | 0.521 | No |
| HexCer 42:1;2O | 0.455 | 0.895 | 0.503 | 0.153 | -0.026 | 0.158 | No |
| HexCer 42:2;2O | 0.370 | 0.344 | 0.177 | 0.348 | 0.373 | 0.447 | No |
| LPC 14:0 | 0.217 | 0.298 | 0.270 | 1.440 | -1.324 | -0.397 | No |
| LPC 14:0 | 0.057 | 0.101 | 0.145 | 0.578 | 0.436 | -0.267 | No |
| LPC 15:0 | 0.478 | 0.418 | 0.938 | 0.203 | 0.142 | -0.019 | No |
| LPC 16:0 | 0.506 | 0.199 | 0.424 | 0.105 | 0.197 | -0.064 | No |
| LPC 16:0 | 0.597 | 0.156 | 0.313 | 0.084 | 0.212 | -0.082 | No |
| LPC 16:1 | 0.084 | 0.254 | 0.581 | 0.326 | 0.214 | -0.101 | No |
| LPC 17:0 | 0.482 | 0.935 | 0.248 | -0.187 | 0.011 | -0.218 | No |
| LPC 17:1 | 0.497 | 0.621 | 0.950 | 0.395 | -0.238 | 0.024 | No |
| LPC 18:0 | 0.090 | 0.752 | 0.045 | -0.313 | 0.040 | -0.328 | No |
| LPC 18:0 | 0.078 | 0.913 | 0.028 | -0.314 | 0.011 | -0.308 | No |
| LPC 18:1 | 0.518 | 0.157 | 0.410 | 0.102 | 0.178 | 0.105 | No |
| LPC 18:1 | 0.458 | 0.175 | 0.995 | 0.114 | 0.160 | -0.001 | No |
| LPC 18:2 | 0.505 | 0.125 | 0.972 | 0.171 | 0.197 | 0.005 | No |
| LPC 18:2 | 0.610 | 0.113 | 0.858 | 0.136 | 0.238 | 0.026 | No |
| LPC 18:3 | 0.371 | 0.025 | 0.573 | 0.353 | 0.704 | 0.178 | No |
| LPC 19:0 | 0.182 | 0.149 | 0.106 | -0.550 | -0.288 | -0.391 | No |
| LPC 20:0 | 0.449 | 0.463 | 0.181 | -0.277 | 0.114 | -0.270 | No |
| LPC 20:1 | 0.288 | 0.061 | 0.266 | -0.345 | -0.249 | -0.227 | No |
| LPC 20:2 | 0.941 | 0.262 | 0.299 | 0.026 | 0.310 | -0.249 | No |
| LPC 20:3 | 0.031 | 0.129 | 0.237 | 0.629 | 0.498 | 0.267 | Yes |
| LPC 20:4 | 0.012 | 0.016 | 0.742 | -0.313 | 0.347 | 0.025 | Yes |
| LPC 20:4 | 0.010 | 0.015 | 0.880 | -0.328 | 0.366 | 0.014 | Yes |
| LPC 22:0 | 0.682 | 0.784 | 0.068 | 0.148 | -0.216 | -0.596 | No |
| LPC 22:1 | 0.643 | 0.733 | 0.202 | -0.202 | 0.123 | -0.353 | No |
| LPC 22:4 | 0.381 | 0.190 | 0.827 | 0.282 | 0.822 | -0.088 | No |
| LPC 22:5 | 0.122 | 0.336 | 0.547 | 0.584 | 0.490 | -0.189 | No |
| LPC 22:5 | 0.761 | 0.163 | 0.435 | 0.075 | 0.493 | -0.197 | No |
| LPC 22:6 | 0.099 | 0.134 | 0.735 | -0.667 | 0.310 | 0.065 | No |
| LPC 22:6 | 0.333 | 0.422 | 0.711 | -0.227 | 0.155 | 0.070 | No |
| LPC 24:0 | 0.933 | 0.041 | 0.694 | 0.021 | -0.448 | -0.074 | No |
| LPC 24:1 | 0.534 | 0.623 | 0.137 | 0.359 | -0.869 | -1.673 | No |
| LPE 16:0 | 0.191 | 0.119 | 0.898 | 0.857 | 0.815 | -0.136 | No |
| LPE 18:0 | 0.779 | 0.938 | 0.144 | -0.144 | -0.072 | 0.759 | No |
| LPE 18:1 | 0.655 | 0.122 | 0.246 | 0.585 | 1.939 | 0.988 | No |
| LPE 18:2 | 0.110 | 0.368 | 0.837 | 1.827 | -1.602 | 0.350 | No |
| PC 30:0\|PC 14:0_16:0 | 0.117 | 0.692 | 0.548 | 0.674 | 0.148 | -0.174 | No |
| PC 31:0\|PC 15:0_16:0 | 0.705 | 0.815 | 0.532 | 0.203 | 0.100 | -0.366 | No |
| PC 32:0 | 0.291 | 0.063 | 0.027 | -0.711 | -1.197 | -1.505 | No |
| PC 32:0\|PC 16:0_16:0 | 0.323 | 0.957 | 0.862 | 0.380 | 0.019 | -0.041 | No |
| PC 32:1\|PC 16:0_16:1 | 0.019 | 0.821 | 0.995 | 1.012 | 0.048 | -0.002 | Yes |

| | | | | | | | |
|---|---|---|---|---|---|---|---|
| PC 32:2\|PC 14:0_18:2 | 0.008 | 0.343 | 0.947 | 1.000 | 0.258 | 0.016 | Yes |
| PC 33:0 | 0.061 | 0.965 | 0.967 | 0.418 | -0.012 | -0.012 | No |
| PC 33:1\|PC 15:0_18:1 | 0.006 | 0.590 | 0.505 | 1.056 | -0.099 | 0.196 | Yes |
| PC 33:2\|PC 15:0_18:2 | 0.096 | 0.789 | 0.355 | 0.585 | -0.058 | 0.299 | No |
| PC 34:0\|PC 16:0_18:0 | 0.500 | 0.790 | 0.306 | 0.136 | 0.042 | 0.136 | No |
| PC 34:1\|PC 16:0_18:1 | 0.004 | 0.830 | 0.238 | 0.763 | 0.028 | 0.233 | Yes |
| PC 34:2\|PC 16:0_18:2 | 0.015 | 0.578 | 0.359 | 0.444 | 0.072 | 0.174 | Yes |
| PC 34:3 | 0.215 | 0.424 | 0.813 | 0.487 | 0.161 | -0.091 | No |
| PC 34:3\|PC 16:1_18:2 | 0.001 | 0.735 | 0.956 | 0.849 | 0.052 | 0.014 | Yes |
| PC 34:4\|PC 14:0_20:4 | 0.029 | 0.345 | 0.305 | 0.777 | 0.338 | -0.339 | Yes |
| PC 35:0 | 0.856 | 0.509 | 0.992 | -0.120 | -0.378 | 0.006 | No |
| PC 35:1 | 0.001 | 0.641 | 0.261 | 0.835 | -0.081 | 0.250 | Yes |
| PC 35:2\|PC 17:0_18:2 | 0.140 | 0.251 | 0.320 | 0.458 | -0.183 | 0.309 | No |
| PC 35:3 | 0.198 | 0.201 | 0.836 | 0.628 | -0.394 | 0.097 | No |
| PC 35:4 | 0.646 | 0.956 | 0.738 | 0.128 | -0.009 | -0.089 | No |
| PC 36:0\|PC 18:0_18:0 | 0.091 | 0.591 | 0.587 | -0.357 | 0.091 | -0.096 | No |
| PC 36:1\|PC 18:0_18:1 | 0.001 | 0.683 | 0.303 | 0.641 | -0.075 | 0.177 | Yes |
| PC 36:2\|PC 18:0_18:2 | 0.019 | 0.663 | 0.393 | 0.463 | -0.050 | 0.137 | Yes |
| PC 36:2\|PC 18:1_18:1 | 0.036 | 0.718 | 0.856 | 0.455 | -0.046 | 0.030 | Yes |
| PC 36:3\|PC 16:0_20:3 | 0.000 | 0.083 | 0.575 | 1.075 | 0.754 | 0.162 | Yes |
| PC 36:3\|PC 16:0_20:3 | 0.001 | 0.454 | 0.360 | 1.993 | 0.267 | 0.389 | Yes |
| PC 36:3\|PC 16:0_20:3 | 0.011 | 0.153 | 0.792 | 0.950 | 0.289 | -0.084 | Yes |
| PC 36:3\|PC 18:1_18:2 | 0.365 | 0.231 | 0.499 | 0.302 | -0.222 | 0.160 | No |
| PC 36:4 | 0.751 | 0.731 | 0.397 | 0.065 | 0.056 | -0.187 | No |
| PC 36:4 | 0.396 | 0.406 | 0.334 | 0.452 | -0.138 | 0.320 | No |
| PC 36:4\|PC 16:0_20:4 | 0.730 | 0.911 | 0.571 | 0.061 | -0.015 | -0.091 | No |
| PC 36:4\|PC 18:2_18:2 | 0.281 | 0.343 | 0.232 | 0.592 | -0.182 | 0.422 | No |
| PC 36:5\|PC 16:1_20:4 | 0.494 | 0.406 | 0.420 | 0.309 | 0.192 | -0.276 | No |
| PC 36:6\|PC 14:0_22:6 | 0.070 | 0.196 | 0.961 | 1.132 | 0.509 | -0.021 | No |
| PC 37:1 | 0.163 | 0.318 | 0.909 | 0.636 | -0.555 | 0.050 | No |
| PC 37:2\|PC 19:0_18:2 | 0.579 | 0.122 | 0.392 | 0.199 | -0.405 | 0.239 | No |
| PC 37:2\|PC 19:0_18:2 | 0.866 | 0.183 | 0.182 | 0.100 | -0.838 | -0.442 | No |
| PC 37:4 | 0.044 | 0.544 | 0.015 | -0.350 | -0.067 | -0.319 | Yes |
| PC 37:4\|PC 17:0_20:4 | 0.357 | 0.123 | 0.330 | -0.300 | -0.243 | -0.198 | No |
| PC 37:5 | 0.007 | 0.733 | 0.934 | 0.971 | 0.187 | -0.027 | Yes |
| PC 37:6\|PC 15:0_22:6 | 0.232 | 0.525 | 0.187 | 0.433 | -0.199 | 0.381 | No |
| PC 38:1 | 0.091 | 0.224 | 0.702 | 0.537 | -0.482 | -0.162 | No |
| PC 38:2\|PC 18:0_20:2 | 0.025 | 0.160 | 0.584 | 0.730 | -0.362 | 0.135 | Yes |
| PC 38:2\|PC 20:0_18:2 | 0.038 | 0.480 | 0.756 | 0.975 | 0.238 | 0.157 | Yes |
| PC 38:3\|PC 18:0_20:3 | 0.007 | 0.566 | 0.900 | 0.722 | 0.114 | 0.027 | Yes |
| PC 38:3\|PC 18:0_20:3 | 0.047 | 0.480 | 0.198 | 0.765 | 0.139 | -0.311 | Yes |
| PC 38:4\|PC 16:0_22:4 | 0.042 | 0.418 | 0.978 | 0.843 | 0.401 | 0.010 | Yes |
| PC 38:4\|PC 18:0_20:4 | 0.072 | 0.131 | 0.076 | -0.342 | -0.173 | -0.263 | No |
| PC 38:5\|PC 16:0_22:5 | 0.429 | 0.752 | 0.503 | 0.325 | 0.093 | -0.175 | No |
| PC 38:5\|PC 16:0_22:5 | 0.043 | 0.694 | 0.809 | 1.063 | 0.174 | -0.078 | Yes |
| PC 38:5\|PC 18:1_20:4 | 0.169 | 0.292 | 0.533 | -0.329 | -0.136 | -0.139 | No |

| | | | | | | | |
|---|---|---|---|---|---|---|---|
| PC 38:6\|PC 16:0_22:6 | 0.611 | 0.586 | 0.502 | 0.136 | -0.107 | 0.136 | No |
| PC 38:6\|PC 18:2_20:4 | 0.753 | 0.538 | 0.501 | 0.066 | 0.104 | 0.173 | No |
| PC 38:7 | 0.551 | 0.504 | 0.716 | 0.149 | 0.167 | -0.119 | No |
| PC 39:5\|PC 17:0_22:5 | 0.028 | 0.425 | 0.705 | 0.606 | -0.337 | -0.137 | Yes |
| PC 39:6\|PC 17:0_22:6 | 0.911 | 0.072 | 0.963 | -0.038 | -0.342 | -0.011 | No |
| PC 39:7 | 0.139 | 0.200 | 0.118 | -0.462 | -0.283 | -0.333 | No |
| PC 40:2 | 0.125 | 0.123 | 0.491 | 0.809 | -0.928 | -0.312 | No |
| PC 40:3 | 0.002 | 0.538 | 0.473 | 2.076 | 0.270 | -0.298 | Yes |
| PC 40:4 | 0.307 | 0.635 | 0.083 | -0.284 | -0.076 | -0.320 | No |
| PC 40:4\|PC 18:0_22:4 | 0.187 | 0.754 | 0.420 | 0.457 | 0.105 | -0.194 | No |
| PC 40:4\|PC 20:0_20:4 | 0.643 | 0.831 | 0.332 | 0.290 | -0.092 | -0.675 | No |
| PC 40:5 | 0.024 | 0.035 | 0.048 | -0.450 | -0.363 | -0.449 | Yes |
| PC 40:5\|PC 18:0_22:5 | 0.102 | 0.525 | 0.054 | 0.774 | -0.182 | -0.411 | No |
| PC 40:5\|PC 18:0_22:5 | 0.553 | 0.267 | 0.046 | 0.147 | -0.159 | -0.357 | No |
| PC 40:6\|PC 18:0_22:6 | 0.652 | 0.083 | 0.156 | -0.122 | -0.384 | -0.147 | No |
| PC 40:6\|PC 20:2_20:4 | 0.017 | 0.691 | 0.234 | 0.507 | 0.182 | -0.445 | Yes |
| PC 40:7\|PC 18:1_22:6 | 0.804 | 0.337 | 0.382 | -0.060 | -0.192 | 0.292 | No |
| PC 40:8\|PC 18:2_22:6 | 0.364 | 0.666 | 0.611 | 0.357 | -0.153 | 0.217 | No |
| PC 40:8\|PC 20:4_20:4 | 0.009 | 0.291 | 0.934 | -0.502 | 0.175 | -0.017 | Yes |
| PC 40:9 | 0.453 | 0.010 | 0.850 | 0.954 | 2.252 | -0.291 | No |
| PC 41:5 | 0.375 | 0.083 | 0.099 | 0.699 | -0.621 | -0.748 | No |
| PC 42:10\|PC 20:4_22:6 | 0.183 | 0.930 | 0.640 | -0.374 | -0.027 | 0.154 | No |
| PC 42:5 | 0.928 | 0.270 | 0.442 | 0.031 | -0.512 | -0.292 | No |
| PC 42:6\|PC 18:0_24:6 | 0.075 | 0.795 | 0.402 | 1.272 | 0.190 | -0.390 | No |
| PC 42:8 | 0.180 | 0.530 | 0.856 | -0.575 | 0.312 | -0.089 | No |
| PC 42:9\|PC 20:4_22:5 | 0.207 | 0.631 | 0.767 | 0.734 | -0.315 | -0.106 | No |
| PC O-32:0\|PC O-16:0_16:0 | 0.603 | 0.596 | 0.972 | 0.144 | -0.141 | -0.008 | No |
| PC O-32:1 | 0.712 | 0.221 | 0.285 | 0.080 | -0.235 | 0.247 | No |
| PC O-32:1 | 0.884 | 0.812 | 0.438 | -0.080 | 0.095 | -0.401 | No |
| PC O-34:0 | 0.362 | 0.993 | 0.595 | 0.442 | 0.005 | -0.308 | No |
| PC O-34:1\|PC O-18:1_16:0 | 0.296 | 0.772 | 0.597 | 0.240 | -0.054 | 0.100 | No |
| PC O-34:2 | 0.160 | 0.914 | 0.712 | 0.379 | 0.026 | -0.068 | No |
| PC O-34:3\|PC O-16:1_18:2 | 0.780 | 0.233 | 0.790 | 0.130 | -1.478 | 0.116 | No |
| PC O-36:3 | 0.248 | 0.739 | 0.504 | 0.335 | 0.116 | -0.196 | No |
| PC O-36:3 | 0.121 | 0.691 | 0.753 | 1.159 | 0.197 | -0.162 | No |
| PC O-36:4\|PC O-16:0_20:4 | 0.346 | 0.935 | 0.726 | 0.202 | -0.012 | -0.074 | No |
| PC O-36:5\|PC O-16:1_20:4 | 0.779 | 0.203 | 0.539 | -0.055 | -0.253 | 0.130 | No |
| PC O-38:4\|PC O-16:0_22:4 | 0.194 | 0.640 | 0.245 | 0.296 | -0.060 | 0.204 | No |
| PC O-38:4\|PC O-18:0_20:4 | 0.217 | 0.724 | 0.582 | 0.267 | -0.052 | -0.117 | No |
| PC O-38:5\|PC O-16:1_22:4 | 0.444 | 0.378 | 0.679 | 0.473 | 0.382 | -0.249 | No |
| PC O-38:5\|PC O-18:1_20:4 | 0.427 | 0.437 | 0.806 | 0.167 | -0.113 | -0.054 | No |
| PC O-38:6\|PC O-16:0_22:6 | 0.437 | 0.596 | 0.488 | 0.325 | 0.098 | 0.206 | No |
| PC O-38:6\|PC O-18:2_20:4 | 0.340 | 0.169 | 0.777 | -0.450 | -0.562 | -0.115 | No |
| PC O-38:7\|PC O-16:1_22:6 | 0.081 | 0.554 | 0.399 | -0.936 | -0.280 | 0.304 | No |
| PC O-39:10 | 0.004 | 0.772 | 0.497 | 0.975 | 0.063 | 0.182 | Yes |
| PC O-40:4\|PC O-20:0_20:4 | 0.263 | 0.377 | 0.008 | 0.446 | -0.251 | -0.929 | No |

| | | | | | | | |
|---|---|---|---|---|---|---|---|
| PC O-40:7\|PC O-18:1_22:6 | 0.658 | 0.530 | 0.904 | 0.351 | -0.165 | 0.067 | No |
| PC O-40:8 | 0.806 | 0.236 | 0.615 | 0.071 | -0.196 | -0.148 | No |
| PC O-41:10 | 0.002 | 0.664 | 0.828 | 0.711 | -0.125 | -0.056 | Yes |
| PC O-41:11 | 0.077 | 0.713 | 0.480 | 0.552 | -0.089 | -0.168 | No |
| PE 34:2\|PE 16:0_18:2 | 0.041 | 0.478 | 0.169 | 2.594 | -0.752 | 0.851 | Yes |
| PE 36:2\|PE 18:0_18:2 | 0.011 | 0.512 | 0.455 | 1.802 | -0.523 | 0.389 | Yes |
| PE 36:4\|PE 16:0_20:4 | 0.066 | 0.758 | 0.907 | 1.059 | -0.125 | 0.046 | No |
| PE 38:4\|PE 18:0_20:4 | 0.085 | 0.782 | 0.703 | 0.768 | -0.136 | -0.109 | No |
| PE 38:5\|PE 18:1_20:4 | 0.258 | 0.652 | 0.279 | 1.362 | -0.303 | -0.655 | No |
| PE 38:6\|PE 16:0_22:6 | 0.131 | 0.449 | 0.632 | 1.385 | -0.468 | 0.276 | No |
| PE 40:6\|PE 18:0_22:6 | 0.586 | 0.227 | 0.952 | 0.162 | -0.412 | 0.016 | No |
| PE O-36:5\|PE O-16:1_20:4 | 0.668 | 0.569 | 0.365 | 0.261 | -0.232 | -0.340 | No |
| PE O-38:5\|PE O-18:1_20:4 | 0.287 | 0.591 | 0.104 | 0.541 | -0.204 | -0.499 | No |
| PE O-38:6\|PE O-18:2_20:4 | 0.266 | 0.398 | 0.361 | 1.106 | -0.529 | -0.518 | No |
| PE P-36:4\|PE P-16:0_20:4 | 0.855 | 0.820 | 0.128 | 0.141 | -0.105 | -0.882 | No |
| PE P-38:5\|PE P-18:1_20:4 | 0.757 | 0.749 | 0.301 | 0.281 | -0.166 | -0.716 | No |
| PI 36:4 | 0.456 | 0.418 | 0.380 | 0.219 | -0.214 | -0.255 | No |
| PI 38:4\|PI 18:0_20:4 | 0.470 | 0.025 | 0.009 | -0.228 | -0.421 | -0.667 | No |
| SE 28:1/20:4 | 0.127 | 0.951 | 0.625 | -1.148 | -0.024 | -0.155 | No |
| SE 29:1/20:4 | 0.082 | 0.945 | 0.506 | -0.859 | 0.028 | -0.264 | No |
| SE 29:2/20:4 | 0.177 | 0.291 | 0.274 | 1.082 | -0.901 | -0.398 | No |
| SM 32:1;2O | 0.382 | 0.455 | 0.350 | 0.375 | 0.186 | -0.276 | No |
| SM 33:1;2O | 0.627 | 0.274 | 0.855 | 0.103 | -0.144 | -0.026 | No |
| SM 34:0;2O | 0.354 | 0.488 | 0.666 | 0.322 | 0.144 | -0.086 | No |
| SM 34:1;2O | 0.877 | 0.624 | 0.511 | 0.041 | 0.078 | 0.104 | No |
| SM 34:1;3O | 0.760 | 0.238 | 0.769 | -0.128 | -0.674 | -0.119 | No |
| SM 34:2;2O | 0.342 | 0.033 | 0.569 | -0.262 | -0.380 | -0.105 | No |
| SM 34:2;3O | 0.638 | 0.139 | 0.126 | 0.337 | -0.754 | -0.973 | No |
| SM 35:1;2O | 0.125 | 0.870 | 0.963 | 0.234 | -0.028 | 0.007 | No |
| SM 36:1;2O | 0.218 | 0.266 | 0.853 | 0.435 | 0.282 | 0.040 | No |
| SM 36:2;2O | 0.725 | 0.327 | 0.091 | 0.072 | -0.180 | -0.260 | No |
| SM 38:1;2O | 0.079 | 0.149 | 0.124 | 0.478 | 0.307 | 0.268 | No |
| SM 39:1;2O | 0.166 | 0.948 | 0.870 | 0.697 | -0.041 | -0.087 | No |
| SM 40:1;2O | 0.182 | 0.109 | 0.112 | 0.232 | 0.257 | 0.202 | No |
| SM 40:2;2O | 0.859 | 0.225 | 0.679 | 0.049 | -0.265 | 0.083 | No |
| SM 40:2;2O | 0.633 | 0.989 | 0.635 | 0.111 | -0.002 | 0.104 | No |
| SM 41:1;2O | 0.972 | 0.299 | 0.364 | -0.006 | -0.183 | 0.100 | No |
| SM 41:2;2O | 0.347 | 0.037 | 0.621 | -0.520 | -0.801 | -0.128 | No |
| SM 41:2;2O | 0.130 | 0.565 | 0.850 | 0.391 | 0.085 | -0.042 | No |
| SM 42:1;2O | 0.693 | 0.772 | 0.717 | -0.057 | -0.037 | 0.037 | No |
| SM 42:2;2O | 0.295 | 0.200 | 0.374 | 0.256 | 0.234 | 0.134 | No |
| SM 42:2;2O | 0.390 | 0.048 | 0.562 | -0.340 | -0.454 | -0.115 | No |
| SM 42:3;2O | 0.462 | 0.508 | 0.920 | 0.162 | 0.141 | 0.015 | No |
| SM 43:1;2O | 0.048 | 0.052 | 0.900 | -1.195 | -0.895 | 0.037 | Yes |
| SM 43:1;2O | 0.832 | 0.330 | 0.495 | -0.059 | -0.270 | -0.222 | No |
| SM 43:2;2O | 0.691 | 0.009 | 0.857 | -0.285 | -1.840 | -0.166 | No |

| | | | | | | | |
|---|---|---|---|---|---|---|---|
| ST 24:1;O4 | 0.042 | 0.589 | 0.016 | 1.768 | 0.255 | 1.536 | No |
| ST 24:1;O4 | 0.318 | 0.430 | 0.583 | 0.792 | -0.611 | 0.446 | No |
| ST 24:1;O4 | 0.556 | 0.149 | 0.142 | 0.667 | 0.598 | 1.455 | Yes |
| ST 27:1;O | 0.150 | 0.665 | 0.935 | 0.346 | -0.094 | 0.020 | No |
| ST 27:1;O | 0.940 | 0.439 | 0.424 | -0.023 | -0.258 | -0.286 | No |
| ST 27:1;O;S | 0.651 | 0.226 | 0.516 | 0.112 | -0.334 | -0.167 | No |
| ST 28:1;O;S | 0.468 | 0.043 | 0.332 | -0.415 | -0.643 | -0.248 | No |
| TG 42:1\|TG 12:0_12:0_18:1 | 0.087 | 0.007 | 0.710 | 0.628 | 0.348 | 0.087 | No |
| TG 44:0\|TG 14:0_14:0_16:0 | 0.052 | 0.320 | 0.948 | 0.440 | -0.124 | 0.008 | No |
| TG 44:1\|TG 12:0_16:0_16:1 | 0.518 | 0.515 | 0.455 | -0.312 | 0.206 | -0.296 | No |
| TG 44:1\|TG 14:0_16:0_14:1 | 0.014 | 0.617 | 0.041 | 0.652 | 0.060 | -0.187 | Yes |
| TG 45:0\|TG 14:0_15:0_16:0 | 0.377 | 0.900 | 0.686 | 0.089 | 0.008 | -0.044 | No |
| TG 45:1\|TG 14:0_15:0_16:1 | 0.328 | 0.600 | 0.778 | 0.119 | 0.070 | 0.023 | No |
| TG 46:0\|TG 14:0_16:0_16:0 | 0.018 | 0.761 | 0.578 | 0.677 | -0.042 | -0.131 | Yes |
| TG 46:1\|TG 14:0_16:0_16:1 | 0.013 | 0.718 | 0.428 | 0.983 | 0.054 | -0.079 | Yes |
| TG 46:2\|TG 12:0_16:0_18:2 | 0.005 | 0.410 | 0.194 | 0.553 | -0.130 | -0.284 | Yes |
| TG 46:3\|TG 12:0_16:1_18:2 | 0.025 | 0.322 | 0.599 | 2.181 | 0.643 | -0.466 | Yes |
| TG 47:0\|TG 15:0_16:0_16:0 | 0.003 | 0.273 | 0.917 | 0.474 | 0.092 | -0.013 | Yes |
| TG 47:1\|TG 15:0_16:0_16:1 | 0.015 | 0.575 | 0.859 | 0.579 | -0.049 | 0.016 | Yes |
| TG 48:0\|TG 16:0_16:0_16:0 | 0.002 | 0.857 | 0.678 | 0.977 | -0.035 | -0.094 | Yes |
| TG 48:1\|TG 14:0_16:0_18:1 | 0.003 | 0.993 | 0.402 | 1.680 | 0.002 | -0.200 | Yes |
| TG 48:2\|TG 14:0_16:0_18:2 | 0.006 | 0.601 | 0.411 | 1.856 | 0.184 | -0.286 | Yes |
| TG 48:4\|TG 12:0_18:2_18:2 | 0.153 | 0.592 | 0.144 | 1.037 | 0.399 | -1.272 | No |
| TG 49:0\|TG 15:0_16:0_18:0 | 0.045 | 0.695 | 0.537 | 0.470 | 0.053 | 0.093 | Yes |
| TG 49:1\|TG 15:0_16:0_18:1 | 0.003 | 0.842 | 0.929 | 1.245 | 0.039 | -0.020 | Yes |
| TG 49:2\|TG 16:0_16:1_17:1 | 0.010 | 0.760 | 0.604 | 1.499 | 0.124 | 0.171 | Yes |
| TG 49:3\|TG 15:0_16:1_18:2 | 0.022 | 0.651 | 0.606 | 1.423 | 0.202 | -0.181 | Yes |
| TG 50:0\|TG 16:0_16:0_18:0 | 0.003 | 0.957 | 0.717 | 0.970 | -0.013 | -0.083 | Yes |
| TG 50:1\|TG 16:0_16:0_18:1 | 0.002 | 0.871 | 0.574 | 1.718 | -0.062 | -0.224 | Yes |
| TG 50:2\|TG 16:0_16:1_18:1 | 0.004 | 0.957 | 0.580 | 1.606 | 0.021 | -0.202 | Yes |
| TG 50:3\|TG 16:0_16:1_18:2 | 0.009 | 0.908 | 0.362 | 1.408 | 0.042 | -0.336 | Yes |
| TG 50:4\|TG 16:1_16:1_18:2 | 0.030 | 0.834 | 0.725 | 1.469 | 0.108 | -0.237 | Yes |
| TG 50:5\|TG 16:0_16:1_18:4 | 0.043 | 0.425 | 0.199 | 1.280 | -0.240 | -0.512 | Yes |
| TG 51:0\|TG 16:0_17:0_18:0 | 0.053 | 0.408 | 0.412 | 0.428 | 0.126 | 0.127 | No |
| TG 51:1\|TG 16:0_17:0_18:1 | 0.002 | 0.921 | 0.953 | 1.471 | -0.026 | -0.018 | Yes |
| TG 51:2\|TG 16:0_17:1_18:1 | 0.006 | 0.924 | 0.985 | 1.438 | -0.027 | -0.006 | Yes |
| TG 51:3\|TG 15:0_18:1_18:2 | 0.027 | 0.753 | 0.798 | 1.220 | -0.116 | -0.116 | Yes |
| TG 51:4\|TG 15:0_18:2_18:2 | 0.039 | 0.880 | 0.937 | 1.002 | 0.076 | -0.049 | Yes |
| TG 52:0\|TG 16:0_18:0_18:0 | 0.037 | 0.850 | 0.726 | 0.512 | 0.036 | 0.060 | Yes |
| TG 52:1\|TG 16:0_18:0_18:1 | 0.002 | 0.949 | 0.998 | 1.925 | -0.022 | -0.001 | Yes |
| TG 52:2\|TG 16:0_18:1_18:1 | 0.008 | 0.453 | 0.882 | 1.049 | -0.284 | 0.062 | Yes |
| TG 52:3\|TG 16:0_18:1_18:2 | 0.059 | 0.561 | 0.835 | 0.686 | -0.183 | -0.078 | No |
| TG 52:4\|TG 16:0_18:2_18:2 | 0.078 | 0.591 | 0.731 | 0.868 | -0.207 | -0.156 | No |
| TG 52:5\|TG 16:0_18:2_18:3 | 0.062 | 0.851 | 0.559 | 0.908 | -0.072 | -0.253 | No |
| TG 52:6\|TG 16:1_18:2_18:3 | 0.046 | 0.672 | 0.101 | 0.900 | -0.118 | -0.748 | Yes |
| TG 53:1\|TG 17:0_18:0_18:1 | 0.004 | 0.873 | 0.577 | 1.218 | -0.049 | 0.163 | Yes |

| | | | | | | | |
|---|---|---|---|---|---|---|---|
| TG 53:2\|TG 17:0_18:1_18:1 | 0.007 | 0.658 | 0.817 | 1.294 | -0.127 | 0.087 | Yes |
| TG 53:3\|TG 17:0_18:1_18:2 | 0.011 | 0.497 | 0.983 | 1.269 | -0.243 | 0.009 | Yes |
| TG 53:4\|TG 17:1_18:1_18:2 | 0.005 | 0.947 | 0.888 | 1.199 | 0.021 | 0.055 | Yes |
| TG 53:5\|TG 16:0_18:2_19:3 | 0.095 | 0.918 | 0.477 | 0.872 | -0.038 | -0.282 | No |
| TG 53:6\|TG 15:0_18:2_20:4 | 0.298 | 0.247 | 0.726 | 0.584 | -0.619 | -0.222 | No |
| TG 54:0\|TG 16:0_18:0_20:0 | 0.041 | 0.398 | 0.460 | 0.259 | 0.085 | 0.079 | No |
| TG 54:2\|TG 18:0_18:1_18:1 | 0.007 | 0.673 | 0.893 | 1.555 | -0.172 | -0.052 | Yes |
| TG 54:3\|TG 18:0_18:1_18:2 | 0.016 | 0.685 | 0.931 | 1.136 | -0.185 | -0.038 | Yes |
| TG 54:4\|TG 16:0_18:1_20:3 | 0.048 | 0.826 | 0.865 | 0.920 | -0.081 | -0.072 | Yes |
| TG 54:4\|TG 18:1_18:1_18:2 | 0.006 | 0.585 | 0.777 | 1.187 | 0.145 | -0.095 | Yes |
| TG 54:5\|TG 16:0_18:1_20:4 | 0.115 | 0.901 | 0.651 | 0.940 | -0.045 | -0.220 | No |
| TG 54:6\|TG 16:0_18:2_20:4 | 0.665 | 0.244 | 0.284 | 0.192 | -0.216 | -0.338 | No |
| TG 54:7\|TG 16:0_18:2_20:5 | 0.340 | 0.790 | 0.023 | 0.391 | -0.058 | -0.493 | No |
| TG 54:7\|TG 18:2_18:2_18:3 | 0.803 | 0.811 | 0.060 | 0.119 | -0.067 | -0.594 | No |
| TG 55:0\|TG 15:0_16:0_24:0 | 0.902 | 0.522 | 0.559 | -0.007 | 0.050 | 0.066 | No |
| TG 55:2\|TG 17:0_18:1_20:1 | 0.113 | 0.912 | 0.609 | 0.919 | 0.047 | 0.211 | No |
| TG 55:3\|TG 19:0_18:1_18:2 | 0.019 | 0.531 | 0.742 | 1.189 | -0.263 | 0.145 | Yes |
| TG 55:4\|TG 17:0_18:1_20:3 | 0.029 | 0.598 | 0.747 | 1.095 | 0.309 | 0.198 | Yes |
| TG 55:5\|TG 17:0_18:1_20:4 | 0.051 | 0.875 | 0.899 | 0.792 | 0.033 | 0.059 | No |
| TG 55:6\|TG 16:0_17:0_22:6 | 0.175 | 0.471 | 0.871 | 0.678 | -0.167 | -0.069 | No |
| TG 56:0\|TG 16:0_16:0_24:0 | 0.054 | 0.687 | 0.436 | 0.144 | 0.048 | 0.080 | No |
| TG 56:1\|TG 16:0_18:0_22:1 | 0.014 | 0.889 | 0.775 | 0.979 | 0.054 | 0.132 | Yes |
| TG 56:2\|TG 16:0_22:0_18:2 | 0.005 | 0.935 | 0.725 | 1.644 | -0.043 | -0.177 | Yes |
| TG 56:3\|TG 16:0_22:1_18:2 | 0.014 | 0.849 | 0.548 | 1.449 | 0.091 | 0.273 | Yes |
| TG 56:4\|TG 16:0_18:1_22:3 | 0.014 | 0.989 | 0.725 | 1.295 | 0.006 | -0.167 | Yes |
| TG 56:5\|TG 16:0_18:1_22:4 | 0.185 | 0.905 | 0.297 | 0.491 | -0.031 | -0.414 | No |
| TG 56:6\|TG 16:0_18:2_22:4 | 0.306 | 0.657 | 0.439 | 0.428 | -0.071 | -0.254 | No |
| TG 56:7\|TG 18:1_18:2_20:4 | 0.758 | 0.660 | 0.668 | 0.145 | 0.093 | -0.168 | No |
| TG 56:8\|TG 18:2_18:2_20:4 | 0.375 | 0.186 | 0.441 | -0.333 | -0.290 | -0.222 | No |
| TG 56:9\|TG 18:2_18:2_20:5 | 0.540 | 0.856 | 0.272 | -0.217 | -0.040 | -0.326 | No |
| TG 56:9\|TG 18:2_18:3_20:4 | 0.804 | 0.439 | 0.002 | 0.123 | -0.207 | -0.764 | No |
| TG 57:0\|TG 16:0_17:0_24:0 | 0.024 | 0.149 | 0.254 | -0.107 | 0.133 | 0.094 | No |
| TG 57:1\|TG 16:0_23:0_18:1 | 0.020 | 0.577 | 0.889 | 0.726 | -0.133 | 0.055 | Yes |
| TG 57:3\|TG 18:1_18:1_21:1 | 0.023 | 0.622 | 0.510 | 1.316 | 0.397 | 0.477 | Yes |
| TG 57:5\|TG 17:0_18:1_22:4 | 0.398 | 0.448 | 0.301 | 0.598 | -0.275 | -0.512 | No |
| TG 57:7\|TG 17:0_18:1_22:6 | 0.210 | 0.380 | 0.631 | 1.110 | -0.270 | -0.223 | No |
| TG 58:0\|TG 16:0_18:0_24:0 | 0.241 | 0.769 | 0.834 | 0.158 | 0.038 | -0.016 | No |
| TG 58:1\|TG 16:0_24:0_18:1 | 0.012 | 0.837 | 0.814 | 0.777 | -0.099 | 0.084 | Yes |
| TG 58:10\|TG 18:2_18:2_22:6 | 0.123 | 0.360 | 0.060 | -1.091 | -0.337 | -0.566 | No |
| TG 58:2\|TG 16:0_18:1_24:1 | 0.026 | 0.657 | 0.831 | 1.161 | -0.256 | 0.118 | Yes |
| TG 58:3\|TG 22:0_18:1_18:2 | 0.035 | 0.699 | 0.903 | 1.192 | -0.244 | 0.076 | Yes |
| TG 58:4\|TG 18:1_22:1_18:2 | 0.098 | 0.714 | 0.827 | 1.083 | -0.227 | 0.168 | No |
| TG 58:6\|TG 18:0_18:1_22:5 | 0.096 | 0.745 | 0.753 | 2.379 | 0.119 | -0.168 | No |
| TG 58:7\|TG 18:1_18:1_22:5 | 0.702 | 0.101 | 0.990 | 0.171 | 0.385 | 0.004 | No |
| TG 58:8\|TG 18:1_18:2_22:5 | 0.842 | 0.496 | 0.299 | 0.094 | -0.188 | -0.258 | No |
| TG 58:9\|TG 18:2_20:3_20:4 | 0.439 | 0.669 | 0.058 | -0.462 | -0.151 | -0.463 | No |

| | | | | | | | |
|---|---|---|---|---|---|---|---|
| TG 60:10\|TG 18:1_20:4_22:5 | 0.664 | 0.923 | 0.695 | -0.285 | -0.025 | -0.120 | No |
| TG 60:11\|TG 18:2_20:4_22:5 | 0.185 | 0.508 | 0.406 | -0.991 | -0.155 | -0.225 | No |
| TG 60:12\|TG 18:2_20:4_22:6 | 0.343 | 0.247 | 0.013 | -0.858 | -0.456 | -0.649 | No |
| TG 60:2\|TG 16:0_20:1_24:1 | 0.030 | 0.848 | 0.705 | 1.090 | -0.129 | 0.235 | Yes |
| TG 60:3\|TG 16:0_26:1_18:2 | 0.038 | 0.587 | 0.851 | 1.164 | -0.372 | 0.121 | Yes |
| TG 60:5\|TG 24:1_18:2_18:2 | 0.026 | 0.802 | 0.685 | 1.700 | 0.242 | -0.217 | Yes |
| TG 60:9\|TG 18:0_20:4_22:5 | 0.693 | 0.200 | 0.870 | -0.325 | 0.334 | -0.038 | No |
| TG 60:9\|TG 18:1_20:4_22:4 | 0.975 | 0.234 | 0.182 | 0.019 | -0.416 | -0.387 | No |
| TG 62:13\|TG 20:4_20:4_22:5 | 0.392 | 0.335 | 0.151 | -1.326 | -0.666 | -0.512 | No |
| TG 62:14\|TG 20:4_20:4_22:6 | 0.987 | 0.569 | 0.408 | -0.041 | -0.271 | -0.363 | No |
| TG 62:2\|TG 18:0_18:1_26:1 | 0.026 | 0.818 | 0.535 | 1.509 | -0.255 | 0.499 | Yes |
| TG 62:4\|TG 18:1_26:1_18:2 | 0.046 | 0.615 | 0.464 | 1.349 | -0.561 | 0.806 | Yes |

**Table S4.** Enrichment analysis results for lipid classes and fatty acid residues.

| Class / Residue | Number of Features | | | *p*-Value | | Enrichment Score | |
|---|---|---|---|---|---|---|---|
| | Total | Increased | Decreased | Increase | Decrease | Increase | Decrease |
| FA 12:0 | 6 | 0 | 0 | - | - | - | - |
| FA 14:0 | 16 | 7 | 0 | 0.634461 | 1 | 0.973536 | 0 |
| FA 15:0 | 17 | 8 | 0 | 0.524319 | 1 | 1.047165 | 0 |
| FA 16:0 | 82 | 50 | 0 | 0.001069 | 1 | 1.356845 | 0 |
| FA 16:1 | 20 | 14 | 0 | 0.019074 | 1 | 1.557658 | 0 |
| FA 17:0 | 18 | 6 | 0 | 0.895631 | 1 | 0.741742 | 0 |
| FA 17:1 | 5 | 0 | 0 | - | - | - | - |
| FA 18:0 | 39 | 17 | 0 | 0.632847 | 1 | 0.96997 | 0 |
| FA 18:1 | 69 | 40 | 0 | 0.013514 | 1 | 1.289986 | 0 |
| FA 18:2 | 76 | 31 | 0 | 0.819999 | 1 | 0.907658 | 0 |
| FA 18:3 | 9 | 1 | 0 | 0.995622 | 1 | 0.247247 | 0 |
| FA 20:0 | 5 | 0 | 0 | - | - | - | - |
| FA 20:3 | 9 | 7 | 0 | 0.047855 | 1 | 1.730731 | 0 |
| FA 20:4 | 51 | 8 | 6 | 0.999999 | 5.93E-06 | 0.349055 | 8.302521 |
| FA 22:0 | 5 | 0 | 0 | - | - | - | - |
| FA 22:4 | 10 | 1 | 0 | 0.997626 | 1 | 0.222523 | 0 |
| FA 22:5 | 18 | 3 | 1 | 0.997807 | 0.230062 | 0.370871 | 3.920635 |
| FA 22:6 | 22 | 0 | 0 | 1 | 1 | 0 | 0 |
| FA 24:0 | 8 | 0 | 0 | - | - | - | - |
| FA 24:1 | 9 | 0 | 0 | - | - | - | - |
| CAR | 4 | 0 | 0 | - | - | - | - |
| CE | 6 | 0 | 2 | 1 | 0.011026 | 0 | 11.36364 |
| Cer | 8 | 1 | 0 | 0.903087 | 1 | 0.49867 | 0 |
| DG | 38 | 10 | 3 | 0.493366 | 0.308382 | 1.049832 | 1.794258 |
| FA | 33 | 0 | 1 | 1 | 0.642172 | 0 | 1.033058 |
| HexCer | 5 | 0 | 0 | - | - | - | - |
| LPC | 31 | 1 | 2 | 0.999916 | 0.228206 | 0.128689 | 2.199413 |
| LPE | 4 | 0 | 0 | - | - | - | - |
| PC | 77 | 24 | 3 | 0.108964 | 0.400539 | 1.243437 | 1.328217 |
| PC-O | 24 | 2 | 0 | 0.992612 | 1 | 0.332447 | 0 |
| PE | 7 | 2 | 0 | 0.558402 | 1 | 1.139818 | 0 |
| PE-O | 3 | 0 | 0 | - | - | - | - |
| PE-P | 2 | 0 | 0 | - | - | - | - |
| PI | 2 | 0 | 0 | - | - | - | - |
| SM | 25 | 0 | 1 | 1 | 0.536804 | 0 | 1.363636 |
| ST | 7 | 1 | 0 | 0.86984 | 1 | 0.569909 | 0 |
| TG | 97 | 53 | 0 | 8.87e-14 | 1 | 2.179754 | 0 |
